# Comparative transcriptomic analysis of human and *Drosophila* extracellular vesicles

**DOI:** 10.1038/srep27680

**Published:** 2016-06-10

**Authors:** Fabio Alexis Lefebvre, Louis Philip Benoit Bouvrette, Lilyanne Perras, Alexis Blanchet-Cohen, Delphine Garnier, Janusz Rak, Éric Lécuyer

**Affiliations:** 1 Institut de Recherches Cliniques de Montréal (IRCM), Montréal, QC H2W 1R7, Canada; 2 Département de Biochimie, Université de Montréal, Montréal, QC H3T 1J4, Canada; 3 Montreal Children’s Hospital, Research Institute of the McGill University Health Center, Montreal, QC H4A 3J1, Canada; 4 INSERM UMR 1064-ITUN; CHU de Nantes, Nantes, 44093, France; 5 Division of Experimental Medicine, McGill University, Montréal, QC H3A 1A3, Canada

## Abstract

Extracellular vesicles (EVs) are membrane-enclosed nanoparticles containing specific repertoires of genetic material. In mammals, EVs can mediate the horizontal transfer of various cargos and signaling molecules, notably miRNA and mRNA species. Whether this form of intercellular communication prevails in other metazoans remains unclear. Here, we report the first parallel comparative morphologic and transcriptomic characterization of EVs from *Drosophila* and human cellular models. Electronic microscopy revealed that human and *Drosophila* cells release similar EVs with diameters ranging from 30 to 200 nm, which contain complex populations of transcripts. RNA-seq identified abundant ribosomal RNAs, related pseudogenes and retrotransposons in human and *Drosophila* EVs. Vault RNAs and Y RNAs abounded in human samples, whereas small nucleolar RNAs involved in pseudouridylation were most prevalent in *Drosophila* EVs. Numerous mRNAs were identified, largely consisting of exonic sequences displaying full-length read coverage and enriched for translation and electronic transport chain functions. By analogy with human systems, these sizeable similarities suggest that EVs could potentially enable RNA-mediated intercellular communication in *Drosophila*.

Although unprotected RNA molecules display short half-lives in biological fluids such as human serum^1^, circulating RNAs can be stabilized in some circumstances, notably via their incorporation within extracellular vesicles (EVs)^2^,^3^,^4^. EV is an umbrella term referring to membrane-delimited nanostructures released by many eukaryotic and prokaryotic cells^5^. Exosomes, an intensively studied class of small EVs (40–100 nm), arise as intraluminal vesicles in endosomal compartments, while microvesicles, or plasma membrane-shed EVs, tend to be larger (100–400 nm) and are released via an actin-dependent abscission process^5^,^6^. Longly dismissed as cellular debris, EVs currently stand as established intercellular shuttles of genetic material, with functional implications particularly salient in immunology^3^,^7^ and cancer biology^2^,^8^. Transcriptomic studies have revealed complex, cell type-specific extracellular RNA (exRNA) repertoires in EVs from diverse biological fluids and cell lines^9^,^10^,^11^. Tumor-shed EVs can transfer functional transcripts to stromal cells and thus remodel the tumor microenvironment^12^,^13^,^14^,^15^. Intercellular shuttling of miRNA activity has been described in diverse systems^13^,^14^. At least one EV-associated long intergenic non-coding RNA (lincRNA) contributes to tumor progression in a hepatocellular cancer model^12^, while the transfer of EVs containing *GFP* mRNA leads to fluorescent protein expression in recipient endothelial cells^16^. Together, these results emphasize the functionality of diverse exRNA biotypes in various mammalian models.

Recently, increasing characterization efforts have extended our appreciation of exRNA repertoires in multiple biological species. For instance, lipid vesicles released by the Gram-negative bacterium *Vibrio cholerae* contain diverse RNAs enriched for intergenic sequences^17^. Exosome-like structures released by the protozoan *Trypanosoma cruzi* encapsulate large transcript populations dominated by short sequences derived from ribosomal RNAs (rRNA) and transfer RNAs (tRNA)^18^. Meanwhile, a comparative analysis of fungal exRNA identified a predominance of small nucleolar RNAs (snoRNA) and tRNAs in *Saccharomyces cerevisiae*, *Candida albicans*, *Paracoccidiodes brasiliensis* and *Cryptococcus neoformans*^19^. The arthropod *Drosophila melanogaster* stands as a key model organism that has enabled discoveries of paramount importance over the last century. In blastoderm *Drosophila* embryos, a high proportion of mRNAs adopts spatially resolved patterns, accumulating near subcellular structures such as plasma membrane domains^20^,^21^. EVs have been implicated in *Drosophila* larval development, where a pool of the Wnt ligand is released from imaginal discs in association with exosomal membranes^22^,^23^, possibly contributing to dissemination of the morphogenic signal and resulting cell fate and body patterning commitments. Although proteomic analyses have identified several novel factors in EVs purified from *Drosophila* cell cultures^24^, the exRNA repertoire remains, to our knowledge, hitherto unexplored. In this comparative study, we used a uniform experimental pipeline to characterize EVs and define exRNA repertoires in two *Drosophila* and two human cell lines. Our morphologic and transcriptomic observations reveal considerable similarities across EVs from these distant metazoan systems: they contain comparable amounts of RNA largely consisting of short ribosomal sequences, retrotransposons, other non-coding RNAs and mRNA signatures enriched for translational functions.

## Results and Discussion

### Size characterization of human and *Drosophila* EVs

We investigated EVs in two *Drosophila* cell lines, Dm-D17-c3 (D17), derived from third instar larvae haltere discs^25^,^26^, and S2R+, a macrophage-like S2 isolate from a late stage embryo primary culture^27^. Both are semi-adherent cell lines expressing hemocyte markers that are characterized by the activation of diverse survival pathways^28^. In contrast to S2R+, D17 cells are highly motile and can form cell-cell junctions^25^. Since human tumor-shed EVs have received considerable attention, we reasoned that inclusion of such models in our analysis along with *Drosophila* samples would provide instructive comparisons. We opted for the EGFR-driven, epidermoid carcinoma line A431^29^ and the highly differentiated hepatocellular carcinoma line HepG2^30^.

**Figure 1:**
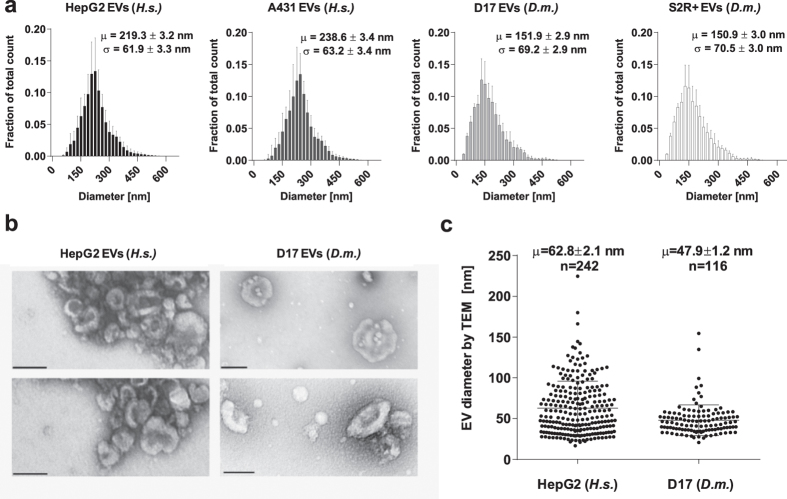
Size distributions of EVs released by human and *Drosophila* cells. **(a**) Histograms depicting the diameter distribution of particles in cell-depleted supernatant, as determined by nanoparticle tracking analyses (Nanosight) for human (*H.s.*) and *Drosophila* (*D.m.*) cells. Mean (μ) and standard deviation (σ) with associated standard error measurements (s.e.m) are overlaid on histograms. **(b**) Representative transmission electron micrographs of human HepG2 (left panels) and *Drosophila* D17 (right panels) EVs purified from culture supernatants and stained with uranyl acetate. Scale bars = 50 nm. **(c**) Whisker plot of diameter distributions of HepG2 (left) and D17 (right) EVs, as determined by direct quantification of electronic micrographs. The number (n) of EVs quantified is indicated.

As a first approach to EV profiling, we performed nanoparticle tracking analyses (NTA) using a Nanosight device on cell culture supernatants cleared of floating cells (30 min at 2000 × g). We calculated the total number of particles found in each preparation based on NTA [μ_HepG2_ = (9.01 ± 1.92) ×10^10^; μ_A431_= (8.80 ± 0.76) ×10^10^; μ_D17_ = (6.72 ± 2.2) ×10^10^; μ_S2R+_ =(1.24 ± 0.35) ×10^11^]. The differences found when comparing HepG2 and A431 EV counts (*P* = 0.84, Mann-Whitney), D17 and S2R+ counts (*P* = 0.057, Mann-Whitney) and all four cell lines (*P* = 0.044, Kruskal-Wallis) were not clearly significant (Figure S1). All samples contained nanostructures of heterogeneous size, displaying characteristic Gaussian distributions (0.87 ≤ R^2^_Gaussian_ ≤ 0.91; Fig. 1A). Particle diameters of the two *Drosophila* lines were not significantly distinct from one another [μ_D17_ = 151.0 ± 2.9 nm; μ_S2R +_ = 150.9 ± 3.0 nm; *P* = 0.97, t-test], while human A431 particles were slightly larger than their HepG2 counterparts [μ_A431_ = 238.6 ± 3.4 nm; μ_HepG2_ = 219.3 ± 3.2 nm; *P* = 0.002, t-test]. Most notably, the mean diameters of human particles were significantly larger than those of *Drosophila* samples (*P* = 0.008, one-tailed t-test). Analysis of cell media supplemented with depleted FBS revealed the absence of contaminant particles. Having established that all four cell models release nanostructures, we performed a standard EV differential ultracentrifugation-based purification. Purified EVs from *Drosophila* D17 and human HepG2 cells were analyzed by transmission electron microscopy (TEM), confirming the prevalence of cup-shaped, exosome-like structures (Fig. 1B). Visual inspection of TEM images suggested that disrupted membrane fragments or protein aggregates were generally absent from the preparations. We took advantage of electron micrographs to carry out comparative manual quantifications of EV diameters (Fig. 1C). *Drosophila* D17 EVs displayed a significantly smaller diameter than human HepG2 EVs (μ_D17_ = 47.9 ± 1.8 nm; μ_HepG2_ = 62.8 ± 2.1 nm, *P* < 10^−4^, t-test). Although the size distributions determined by NTA and TEM partially overlapped, we observed a slight discrepancy when comparing the two techniques, with NTA pointing at larger average size than TEM. Indeed, small EVs (<50nm) were abundant in micrographs of HepG2 and especially D17 EVs, but NTA profiles were largely exempt of bodies smaller than 75 nm. It should be noted that uranyl acetate staining can alter EV morphology, likely causing EVs to collapse and present smaller diameter than what would be observed in solution^31^. Furthermore, aggregates formed by several EVs may be interpreted as single particles by the Nanosight instrument, leading to an overestimation of EV diameter distributions in NTA results. We sometimes observed aggregated EVs during TEM analyses in spite of extensive agitation, a feature that likely arises as a consequence of the ultracentrifugation procedure. Aggregation appeared more prevalent in the case of HepG2 than D17 EVs, which may underlie inherent differences in surface properties of EVs, possibly mediated by the expression of specific membrane proteins. HepG2 cells are considerably more adhesive than D17 cells, which are easily detached from plastic surfaces without trypsin. Whether EVs mirror the adhesion properties of their parental cell type is an interesting and seemingly unexplored question.

Discrepancies between NTA and TEM could thus result from morphological alterations induced by sample preparation for TEM, errors in NTA underlying aggregation, inherent limitations of each technique or a combination of these factors. Previous studies have relied on cryo-electron tomography (ET)^32^ to circumvent the artifacts associated with heavy metal staining while assessing aggregation and derive reliable estimates of tridimensional EV diameter. In a future study, it would be interesting to systematically contrast NTA results with TEM and ET estimates of *Drosophila* EV size to yield a more robust comparison. Nonetheless, our TEM and NTA results both indicate that *Drosophila* D17 EVs are smaller than human HepG2 EVs.

### Human and *Drosophila* EVs enclose complex populations of protected small RNAs

We extracted and quantified protein and DNAse-treated RNA from biological triplicates of *Drosophila* S2R+ and D17 EVs, in conjunction with human HepG2 and A431 EVs. We found that all EV samples, collected over a 48 h window, contained ~100–250 μg of protein and ~200–650 ng of RNA (Figure S2). No significant difference was found between the variance of these distributions (*P* = 0.11 and *P* = 0.91, ANOVA). We took advantage of total EV counts determined by NTA and attempted to infer estimates of total protein and RNA mass per EV. This effort was motivated by recent reports showing that miRNA counts per EV are very low, less than one copy per EV on average, raising doubts about the potential functional impact of EV-mediated miRNA transfer^33^. Although the precision of our quantifications are limited by the biases of NTA count estimations, the sensitivity of photometric biomolecule quantification and the inherent heterogeneity of EV size and composition, we found that individual EVs isolated from all cell lines contain ~1–3 femtogram/fg (10^−15^ g) of protein [μ_HepG2_ = 2.61 ± 0.74 fg; μ_A431_ = 2.37 ± 34 fg; μ_D17_ = 2.17 ± 0.77 fg; μ_S2R + _ = 1.29 ± 0.42 fg]. Our calculations indicate that S2R + EVs contain significantly more protein (*P* = 0.0045, Mann-Whitney) than D17 EVs. Individual RNA quantification revealed lower values in the range of attograms/ag (10^−18^ g) that displayed strong variability [μ_HepG2_ = 5.71 ± 2.60 ag; μ_A431_ = 5.83 ± 3.07  ag; μ_D17_ = 4.26 ± 2.07 ag; μ_S2R +_ = 2.57 ± 1.20 ag] (Figure S3). Arithmetic estimates indicate that 1.0 ag of a single-stranded RNA accounts for approximately 37 copies of a molecule containing 50 nt, or 3.7 copies for a 500 nt-long molecule^34^. Although our experimental setup doesn’t enable estimations of specific RNA molecule counts, these NTA results are consistent with the presence of a few dozens of RNA molecules in an average EV for all cell types considered. Whether such amounts are sufficient to drive functional changes in recipient cells upon *in vivo* EV transfers remains unclear.

We next performed an RNAse protection assay, where fresh EV pellets were split in two equal parts, either submitted to an RNAse A treatment followed by RNA extraction or a direct RNA extraction. Only a minor proportion of exRNA was degraded upon RNAse A treatment in all 4 EV types, a difference that was not statistically significant, while parallel treatments of total RNA extracted from HepG2 and D17 cells led to complete degradation (Figure S4). These results are consistent with a topological exclusion of exRNA from the surrounding solution by intact EV lipid membranes. We then conducted bioanalyzer capillary electropheresis on all exRNA and total cellular RNA types to compare their size distributions. Small species ranging in size from 50–250 nt were most prevalent in all exRNA electrophoretic profiles, although signatures of longer transcripts were also present (Figure S5). Human and *Drosophila* exRNA profiles were highly similar and mature 18S and 28S peaks were largely absent of all preparations, in accordance with previous reports^2^,^3^,^19^,^30^.

### Ribosomal RNA and related pseudogenes are predominant in human and *Drosophila* EVs

Morphological and structural similarities shared between human and *Drosophila* EVs led us to investigate exRNA repertoires in both species. We performed RNA-seq on duplicates of *Drosophila* D17 and S2R + exRNA, along with human HepG2 and A431 exRNA. To provide unaltered portrayals of exRNA, we chose not to perform any selection or depletion procedure prior to sequencing. To enable comparisons with matching cell transcriptomes, we analyzed duplicates of rRNA-depleted libraries from HepG2 and D17 cellular RNA in parallel. While most previous transcriptomic studies examining EVs have opted to sequence the small RNA fraction, we chose to generate standard sized libraries, which should capture the bulk of EV RNAs as determined by our bioanalyzer profiles (Figure S5). Chiefly, it should emphasize protein-coding and long non-coding RNA repertoires, which have received less attention than EV-associated miRNAs and would typically not be traced in the small RNA fraction. However, our approach cannot emphasize the presence of transcripts shorter than 50 nt, such as mature miRNAs, which would require the preparation of small RNA-seq libraries.

Various rRNA sequences were predominant in all exRNA libraries (Table 1). Pseudogenes derived from 5S rRNA, such as human *RNA5SP145* and *RNA5SP149*, were especially abundant (Figure S6), along with the mitochondrial *RNR1* (12S) and *RNR2* (16S) rRNAs (Figure S7). Full-length bidirectional read distributions mapped to these loci in human EVs and similar species prevailed in *Drosophila* exRNA (Table S1). The 28S and 18S ribosomal RNAs were the single most abundant sequences in all samples. This observation is in sharp contrast with the scarcity of the corresponding ~2 and ~3 kb peaks on EV bioanalyzer imprints. Such apparent inconsistency could underlie the cleavage of EV-targeted 18S and 28S to yield shorter transcripts. Indeed, although read coverage for most transcripts in EVs mimicked corresponding cellular signatures, our analyses can’t ensure that they correspond to full-length RNAs. Since we sequenced 50 nt-long paired-end reads, arrays of cleaved RNA fragments could have potentially resulted in similar coverage signatures. Ribosomal RNA turn-over remains poorly characterized and while the 18S and 28S rRNAs are associated with longer cytoplasmic half-lives than polyadenylated RNAs, rRNA stability in fibroblasts is considerably affected by growth conditions^35^. It is tempting to speculate that EV targeting could serve as a selective clearance mechanism for rRNAs, a view that would be compatible with fragmentation of full-length 18S and 28S. Abundance of rRNAs in EVs has been reported in some systems, notably human serum and urine samples^36^, although most studies have rather outlined the absence of long rRNAs in EVs^3^,^10^. These differences likely reflect both inherent specificity of the model considered and contrasting experimental strategies (eg. small vs long RNA libraries). In some models, apoptotic bodies and microvesicles have been reported to contain higher proportions of rRNAs than exosomes^10^. While several large structures (>100 nm) seen on our TEM micrographs may constitute microvesicles, apoptotic bodies should be rare in our preparations, since we routinely monitored cell death before our experiments and consistently observed rates lower than 5% in all cultures.

Besides hypothetical fragmentation, ambiguous mapping issues may also account for the discrepancy between bioanalyzer profiles and abundant 18S and 28S sequencing reads. Indeed, we found two poorly characterized rRNA-like short sequences (<200 nt) that were highly abundant in human EVs and relatively rare in cells, *AC079949.1* and *AL161626.1*, annotated as “novel miRNAs” in Ensembl and as “rRNA pseudogenes” in GATExplorer. These miRNA loci overlap with large ribosomal subunit repeats^37^ (Figure S8), suggesting that reads arising from these species or similar rRNA-related sequences could potentially have been mapped to 28S genes by read alignment tools, inflating the proportion of rRNA reads in EVs. *AC079949.1* and *AL161626.1* were first identified in human EV extracts and are mentioned in a patent request regarding the use of mesenchymal stem cell-derived EVs for wound therapy^38^. Asymmetric and bidirectional read distributions consistent with an abundant sense-antisense RNA pair were observed for *AC079949.1* in exRNA (Figure S8). Such sense-antisense pairs can anneal and form double-stranded RNA, initiating interference pathways through Dicer activation^39^,^40^. While the functional role of *AC079949.1* remains to be determined, the contribution of short transcripts to rRNA regulation is demonstrated in mammalian cells^41^. Moreover, it is becoming clear that diverse mammalian tissues express “specialized” ribosomes that bear diverse rRNA, ribosomal proteins and isoform specificities^42^. EV transfer could potentially modulate recipient cell ribosomal repertoire, perhaps through phenocopying of donor cell rRNAs. It is thus tempting to speculate that the abundance of rRNA-related “novel miRNA” species in EVs reflects an additional layer of intercellular regulation in ribosomal biogenesis or translational fine-tuning.

### exRNA distributions correlate across cell types in human and *Drosophila*

**Figure 2:**
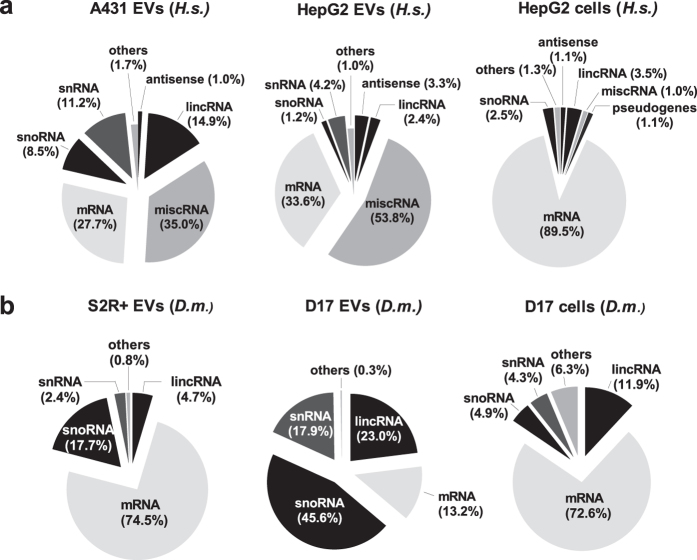
Human and *Drosophila* EVs enclose various types of transcripts. **(a,b)** Pie chart depictions of biotype abundances for the 1,000 most abundant transcripts identified by RNA-seq in human (A431 and K562) (**a**) and *Drosophila* (S2R+ and D17) (**b**) cells lines and their derived EVs. Biotype relative abundance was determined on the basis of TPM values. Reads mapping to rRNA were excluded from this analysis. Biotypes associated with values inferior to 0.5% were grouped into the “others” category.

To further characterize EV RNA repertoires, we next subtracted rRNA reads and submitted adjusted libraries to genomic alignments and expression analyses (Table 2). We retrieved GENCODE annotations using Ensembl Biomart for the 1,000 most abundant transcripts in each library and compared exRNA and cellular RNA biotype abundance distributions (Fig. 2). Interestingly, a strong positive correlation was observed between human HepG2 and A431 exRNA biotypes (Fig. 2A; Pearson’s r = 0.92, *P* = 3 × 10^−3^). The miscellaneous RNA category was strongly overrepresented in human exRNA, accounting for over one third of A431 and over half of HepG2 exRNA distributions, as opposed to 1% for HepG2 cell RNA. In *Drosophila* D17 EVs, snoRNAs represented nearly half of the distribution, whereas S2R + EVs contained a majority of mRNAs (Fig. 2B).

**Figure 3:**
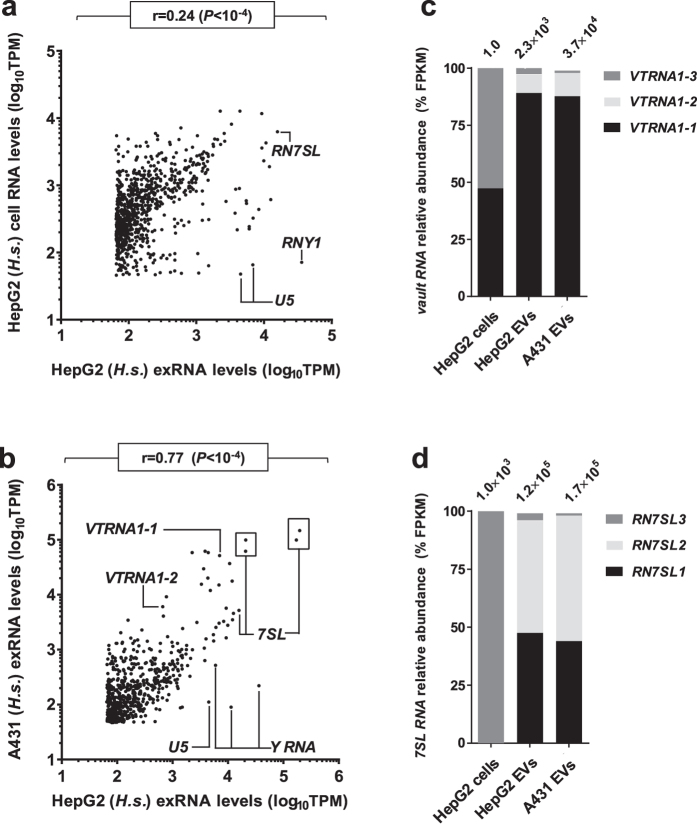
Correlative comparisons of exRNA secreted by human cell lines. **(a,b)** HepG2 exRNA levels were compared to HepG2 cellular RNA (**a**) and A431 exRNA (**b**) levels. Pearson’s correlations (r) and associated p-values are indicated at the top of each graph. Select groups of transcripts are identified. **(c,d)** Bar charts representing the distributions of sequencing reads mapped to paralogues of vault RNAs (**c**) and 7SL RNAs (**d**) in human EVs and cells. Values at the top of each column refer to the total number of reads mapped to these transcripts.

**Figure 4:**
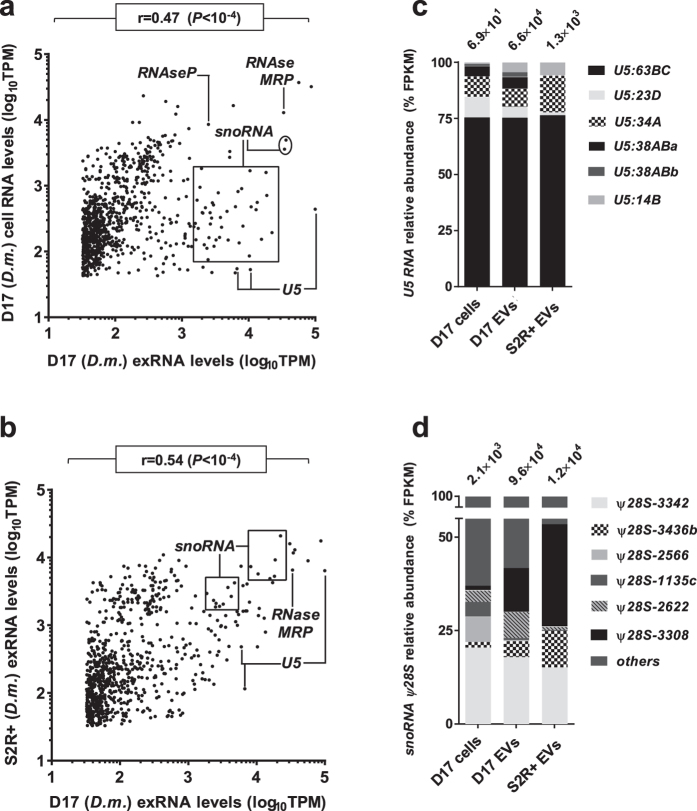
Correlative comparisons of exRNA secreted by *Drosophila* cell lines. **(a,b)** D17 exRNA levels were compared to D17 cellular RNA (**a**) and S2R + exRNA (**b**) levels. Pearson’s correlations (r) and associated p-values are displayed at the top of each graph. Select groups of transcripts are identified. **(c,d)** Bar charts representing the distribution of sequencing reads mapped to lncRNAs (**c**) and snoRNAs (**d**) in *Drosophila* EVs and cells. Values at the top of each column refer to the total number of reads mapped to these transcripts.

We then compared EV and cellular RNA libraries by correlating relative abundance values of individual transcripts (Figs 3 and 4). Although many abundant cellular RNAs were present within EVs, HepG2 exRNA levels were poorly correlated to cellular levels (Fig. 3A; Pearson’s r = 0.24, *P* < 10^−4^) and more closely resembled A431 exRNA levels (Fig. 3B; Pearson’s r = 0.77, *P* < 10^−4^). In accordance with previous reports, three groups of “miscellaneous” Polymerase III products^43^ were strongly overrepresented in human exRNA samples: vault RNAs, Y RNAs and signal recognition particle components, 7SL RNAs. Interestingly, vault RNAs were nearly absent of HepG2 cellular libraries, while the relative levels of vault paralogues was nearly identical in A431 and HepG2 EVs (Fig. 3C). Although the vault ribonucleoprotein (RNP) complex has been linked to antineoplastic drug resistance^44^,^45^,^46^, the bulk of vault RNA transcripts do not associate with this RNP complex^47^. A fraction is rather processed by Dicer into miRNA-like regulatory transcripts that can downregulate the catabolic cytochrome CYP3A4^48^. While *VTRNA1-1* shows full-length coverage in exRNA (Figure S9), its eventual intercellular transfer could potentially impact xenobiotic metabolism in recipient cells. In addition, a strong dissymmetry was noted among 7SL paralogue distribution : *RN7SL3*, the primary detectable cellular paralogue, was barely present in exRNA, while *RN7SL1* and *RN7SL2* accounted for over 90% of total 7SL abundance in exRNA and were undetected in HepG2 cells (Fig. 3D). Multiple sequence alignments^49^ revealed that *RN7SL1* and *RN7SL3* have nearly identical sequences (97.6% identity score). Presumably, the few nucleotides that distinguish these paralogues lead to the establishment of differential interactions resulting in extensive EV targeting of *RN7SL1/2* and cellular retention of *RN7SL3*.

In accordance with human data, *Drosophila* D17 exRNA levels presented lower correlations with corresponding cellular levels (Fig. 4A; Pearson’s r = 0.45, *P* < 10^−4^) than with S2R + exRNA (Fig. 4B; Pearson’s r = 0.58, *P* < 10^−4^). Multiple snoRNAs were overrepresented in D17 exRNA samples, especially H/ACA box species involved in site-specific pseudouridylation of 18S and 28S rRNA (Table S2). Components of atypical snoRNPs were also abundant in human and *Drosophila* exRNA, notably human *RPPH1* and *Drosophila RNAseP:RNA*, which function in tRNA maturation^50^. The human and *Drosophila* transcripts of RNAse MRP, a multifunctional ribozyme notably involved in 5.8S rRNA processing^51^, were abundant in both exRNA and cellular RNA, along with diverse paralogues of spliceosomal U5 snRNA (Figure S10). The *Drosophila CG13900* gene, which encodes a putative spliceosomal factor^52^, contains 9 snoRNAs within its introns, two of which were highly abundant in exRNA (Figure S11). Read coverage at this locus strongly suggests that mRNAs and associated intronic snoRNAs constitute independent transcriptional units, with divergent fates regarding their incorporation into EVs. We also observed low levels of diverse tRNAs in human and *Drosophila* EVs (Figure S12). Several, but not all *Drosophila* snoRNAs and tRNAs, displayed asymmetric read coverage relative to corresponding cells, similar to the pre-miRNA *AC079949.1* discussed above. Such patterns are intriguing in light of accumulating evidence of miRNA-like transcripts derived from tRNAs and snoRNAs involved in RNA interference pathways^53^,^54^,^55^. Indeed, a recent study found that most small RNAs in mature mammalian sperm correspond to 3′-end fragments of tRNAs (tsRNAs), which may modulate cholesterol metabolism in the offspring^56^. Whether snoRNAs and tRNAs that encode miRNA-like substrates are preferentially targeted to EVs remains unclear, but has been suggested^57^. Investigating snoRNAs and tRNAs after selecting for the small RNA population of *Drosophila* EVs may provide further interesting observations regarding tsRNAs.

### Transposable elements are conserved EV components in human and *Drosophila*

Transposable sequences such as long interspersed elements (LINEs) and *Alu* elements have been described as major components of exRNA, notably in glioblastoma models^9^,^58^. We took advantage of the RepeatMasker inventory of interspersed repeats and low complexity sequence genomic coordinates to systematically survey repeats in exRNA^59^. Alignments revealed that repeats are collectively overrepresented in exRNA relative to cellular RNA samples, especially in *Drosophila* (Table 2). Interestingly, the single most abundant repeat sequence in *Drosophila* D17 exRNA was a short (~150 nt) antisense fragment of the retrotransposon *TART*, a telomere-specific LINE-like element involved in chromosome length maintenance^60^ (Figure S13). Upon DNA replication, yeast and mammalian cells depend on the reverse-transcriptase activity of the telomerase complex to regenerate G-rich repeats and assemble the end of chromosomes^61^,^62^,^63^. Drosophilids rely on retrotransposition of telomeric LINE-like elements as an alternative solution to the end-replication problem^64^,^65^,^66^. Other *Drosophila* retrotransposons, notably *Copia* and related sequences were also highly abundant. This 5 kb-long element displayed full-length coverage in S2R + and D17 exRNA (Figure S14). Interestingly, as observed in the case of *TART*, a resolved antisense peak of approximately 50 nt mapped to the central region of the *Copia* sequence in D17 and S2R + exRNA. Repeat-associated small interfering RNA (rasiRNA) form a class of Piwi-interacting transcripts involved in transposable element silencing during *Drosophila* gonadogenesis^67^,^68^,^69^,^70^. Whether rasiRNAs are present in *Drosophila* exRNA is unclear, but it is tempting to suggest that the short antisense sequences mapped to *Copia* and *TART* in exRNA may function as small interfering RNA precursor.

Human endogenous retrovirus long terminal repeats, notably members of the ERV1, ERVL and ERVK subfamilies were the most abundant repeats in HepG2 and A431 exRNA. A few full-length L1 retrotransposons, several Alu, 7SL and tRNA repeats were also prevalent in human exRNA (Table S3). By contrast, α-satellite sequences and low-complexity repeats such as poly-purine or poly-pyrimidine tracts were rare in human exRNA samples. While evidence remains scarce, it is tempting to speculate that extensive EV targeting of retroviral-like sequences constitutes an adaptation that promotes invasion via horizontal transfers.

### Human and *Drosophila* exRNA contain full-length mRNA signatures enriched for translation-related functions

**Figure 5:**
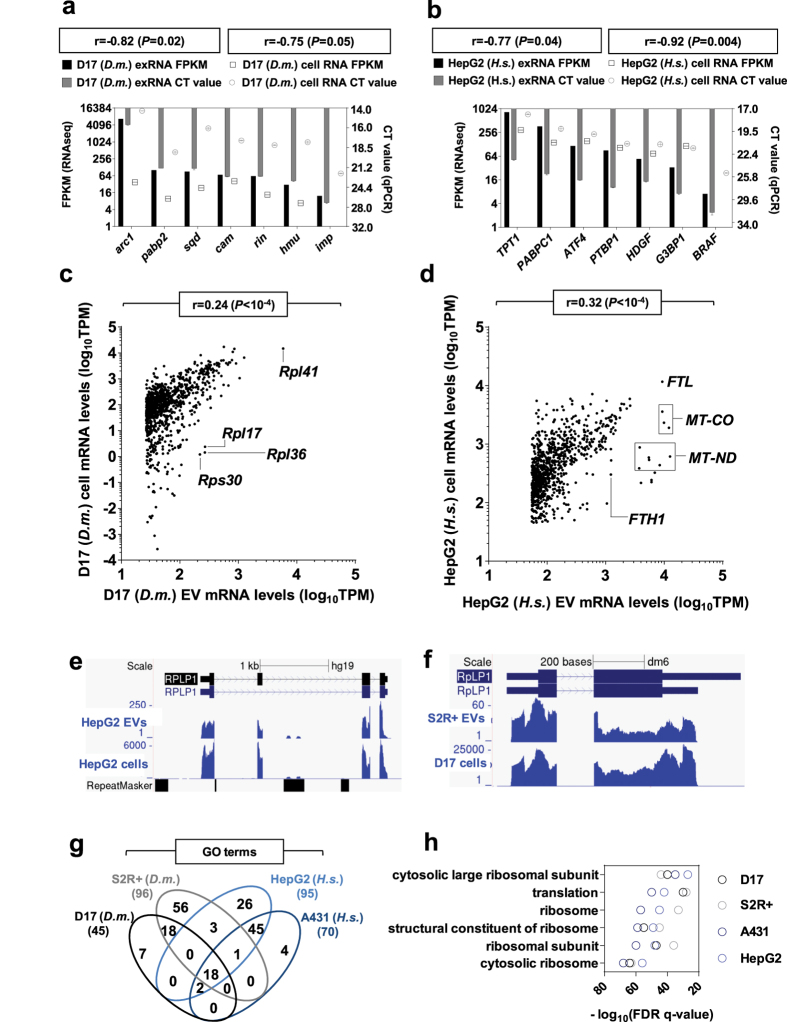
Characterization of mRNAs secreted within Human and *Drosophila* EVs. **(a,b)** Comparative analysis of expression levels of select mRNAs via RNA-seq and qRT-PCR. Cycle threshold (CT) values determined by qRT-PCR are negatively correlated to FPKM values determined by RNA-seq for various *Drosophila* D17 (**a**) and human HepG2 (**b**) mRNAs in EVs and cells. **(c,d)** Relative expression levels of mRNAs extracted from D17 (**c**) or HepG2 cells and EVs (**d**). Select groups of transcripts are identified. Pearson’s correlations (r) and associated p-values are indicated at the top of each graph. **(e,f)** UCSC genome browser views of *Rplp1* mRNA shows strictly exonic read coverage in human HepG2 (**e**) and *Drosophila* D17 (**f**) EVs and cells. **(g)** Venn diagram depicting the overlap of enriched gene ontology (GO) terms displayed for human and *Drosophila* EVs mRNAs. The number of enriched GO terms retrieved per sample is shown. **(h)** Examples of translation-related GO terms identified in all EV samples. Associated false discovery rates (FDR) are provided.

Over 1,000 mRNA signatures were traced in all exRNA samples. We validated RNA-seq results by RT-qPCR analyses targeting 7 mRNAs in exRNA and cellular RNA of *Drosophila* D17 and human HepG2 samples. As expected, FPKM and cycle threshold values (CT) were significantly anti-correlated (−0.92 ≤ Pearson’s r ≤ −0.75) (Fig. 5A,B). Importantly, D17 exRNA displayed low amounts of mRNAs, which were more abundant in S2R + exRNA (Fig. 2B) and mRNA level comparisons between D17 EVs and cells revealed a weaker correlation than global comparisons (Fig. 5C; Pearson’s r = 0.24, *P* < 10^−4^). By contrast, human HepG2 mRNA levels in EVs were closer to corresponding cell levels than described above for global comparisons in human HepG2 (Fig. 5D; Pearson’s r = 0.32, *P* < 10^−4^). Accordingly, several highly expressed cellular mRNAs were traced in EVs, notably mitochondrial mRNAs involved in the respiratory chain, such as cytochrome oxidase, NADH:ubiquinone reductase and ATPase subunit mRNAs (Table S4). Numerous ribosomal protein and translation elongation factor mRNAs were also abundant in all samples. Protein-coding transcripts typically displayed full-length read coverage in cells and EVs, consistent with the EV export of mature and potentially functional mRNAs, as observed for the ribosomal gene *RPLP1* in human and *Drosophila* (Fig. 5E–F). Gene ontology enrichment analyses identified 18 terms common to all four exRNA types, 12 of which contain the words “translation” or “ribosome” (Fig. 5G,H and Table S5). In addition, the ferritin light chain transcript was among the most abundant mRNAs in human HepG2 and A431 exRNA. Ferritin proteins assemble into large shell-like complexes that enclose and store iron ions^71^. These proteins have been identified in EVs of immune origin from mice and human models^72^,^73^,^74^. Interestingly, another important iron regulator, the transferrin receptor, figures among the first factors described in human EVs and its release from reticulocytes is linked to their maturation into erythrocytes^75^,^76^,^77^. EV targeting of these mRNAs and encoded proteins may reflect an intricate layer of iron metabolism regulation. The most abundant mRNA in *Drosophila* D17 exRNA was *Arc1* (*Activity-regulated cytoskeletal protein 1*), orthologous to a mammalian plasticity protein involved in synaptogenesis^78^. Indeed, mammalian *Arc* mRNA is targeted to rat neuronal dendrites via a *cis*-regulatory motif found in its 3′UTR^79^. If this mechanism is conserved in *Drosophila*, it could promote *Arc1* accumulation to plasma membrane domains, resulting in its preferential incorporation within EVs.

Whether a subset of cellular mRNAs undergoes selective targeting to EVs through a sequence-specific mechanism remains unclear. If specific RNA motifs are involved in sorting mRNAs to EVs and if such motifs are conserved, a fraction of orthologous human and *Drosophila* sequences should exhibit a common propensity to accumulate in EVs. To test this hypothesis, we took advantage of our vast EV repertoire of human and *Drosophila* mRNAs to investigate global abundance correlations across orthologs of the two species. Using an integrative ortholog prediction tool^80^, we retrieved 1,140 pairs of orthologous mRNAs represented in our D17 and HepG2 cellular and EV datasets. We first compared cellular relative abundance values, which revealed a strong positive correlation (Figure S15; Pearson’s r = 0.61, *P* < 10^−4^). These distributions were largely dominated by abundant mRNAs encoding ribosomal proteins. When comparing the relative abundance distributions of orthologs in EV, we documented a slightly weaker correlation (Figure S15; Pearson’s r = 0.54, *P* < 10^−4^). Therefore, while the expression levels of orthologous mRNAs are strongly related, our comparative analysis does not suggest that gene-specific mRNA enrichment to EVs is a globally conserved feature.

Previous comparative studies have identified so-called “exRNA-exclusive” transcripts, likely undetected in cognate cells due to highly efficient subcellular transport processes, high cellular turnovers or a combination thereof^81^. To systematically and stringently survey exRNA exclusive transcripts, we compared exRNAs reaching a 5 FPKM threshold to all transcripts detected in cognate cellular libraries. Interestingly, several of the 24 “exRNA-exclusive” *Drosophila* mRNAs encode neuronal membrane-associated proteins, notably *Snap25* (Synaptosomal-associated protein)^82^, along with extracellular matrix factors, such as members of the mucin family (*Muc26B*, *Mu4B*)^83^ (Table S6). The functional relevance of expressing neuronal factors in haltere disc cells is unclear and extensive EV targeting of corresponding mRNAs could constitute a strategy to clear transcriptional noise products and prevent aberrant protein expression. This interpretation is in line with the membrane protein clearance function of EV targeting, well established during reticulocyte differentiation to erythrocytes^76^. Considering the prevalence of mRNA localization and spatially restricted translation during *Drosophila* embryogenesis^21^,^84^, it also appears conceivable that functional cis-regulatory RNA elements present in these sequences promote their targeting to membrane domains, as suggested above for *Arc1* mRNA. Among the 5 exRNA-exclusive human HepG2 mRNAs, *ALB* (*Albumin*) and *APOB* (*Apolipoprotein B*) encode secreted proteins involved in transmembrane transport^85^,^86^ while *TSPN16* (*Tetraspanin 16*) belongs to a class of membrane-spanning factors described as EV protein markers^85^,^86^. In line with our findings in *Drosophila*, the exclusivity of these mRNAs within EVs suggests the prevalence of instructive targeting signals in their sequence that promote local translation of protein factors associated with membrane or extracellular localization.

## Conclusion and Perspectives

To the best of our knowledge, we provide here the first morphological and transcriptomic comparative analysis of human and *Drosophila* EVs. Our work revealed that several features of EVs are considerably conserved in these distant metazoan species, notably the abundance of ribosomal sequences and retrotransposons, including sense-antisense RNA pairs. *Drosophila* EVs released by S2R+ and D17 cells are enriched for diverse snoRNAs, while human EVs produced by HepG2 and A431 cells contain strong signatures of miscellaneous RNAs, such as vault and Y RNAs. While EM and NTA analyses have shown considerable heterogeneity in our preparations regarding EV size and morphology, transcriptomic analyses were performed on EV populations, and therefore can’t determine whether individual EVs display disparate RNA repertoires. Diverse methods such as immunoaffinity captures or density-based separation have been developed to isolate exosomes from preparations containing larger vesicles^87^, and it would be interesting to optimize these tools for *Drosophila* EVs. Previous studies have suggested that mammalian exosomes and microvesicles may contain contrasting molecular signatures, with exosomes showing higher amounts of RNA than microvesicles^10^. Approaches amenable to single EV capture and RNA sequencing have yet to be developed due to the prohibitively small size of these structures, but would be highly appealing since they may reveal the heterogeneity of EV RNA repertoires, notably in *Drosophila*. Furthermore, the use of long-read RNA sequencing platforms^88^, such as single-molecule real-time sequencing (15,000 bp per read) or pyrosequencing (700 bp per read), represent a interesting avenue to further characterize exRNA populations. Such approaches would help illuminate the precise repertoire or RNA isoforms and fragmentation intermediates present within EV specimens. Refined means of intercellular communication have emerged and expanded throughout the evolutionary history of metazoans. While exRNA shuttling likely contributes to cell-cell signaling in mammals, prevalence and functional relevance of the phenomenon remains unexplored in *Drosophila.* In light of our findings, it seems likely that diverse *Drosophila* lineages, in line with their human counterparts, rely on exRNA to convey intercellular communication. This hypothesis could be explored via loss-of-function studies.

## Materials and Methods

### Cell culture and EVs purification

Human HepG2 and A431 cells were maintained in Dulbecco’s Modified Eagle Medium (DMEM) supplemented with 1% of a 1:100 solution of penicillin and streptomycin (pen/strep) and 10% fetal bovine serum (depleted FBS) (Wisent). Before use, FBS was depleted of bovine EVs by ultracentrifugation (110,000 g, 18 h, 4 °C). Human cells were cultured at 37 °C, in an atmosphere of 5% CO_2_ in T-flasks of 175 cm^2^ and routinely detached using 0.25% trypsin upon reaching 80% confluence. *Drosophila* D17 and S2R+ cells were respectively maintained in Shield and Sang Insect Medium (M3) and in Schneider medium. Both media were supplemented with 1% of a 1:100 pen/strep solution and 10% depleted FBS. M3 medium additionally contained insulin (20 μg/mL). *Drosophila* cells were cultured at 25 °C. Cell death rate was routinely monitored by trypan blue staining (Sigma-Aldrich) and consistently remained below 5% for all cell types.

### Isolation of EVs

For EV isolation, cells were kept at low passage (P < 10) and cultured for approximately 48 h starting from ~10^7^ cells. EVs were isolated according to an established differential ultracentrifugation protocol^89^. Briefly, fresh culture supernatants (~80 mL) were cleared of floating (10 min at 400 × g) and dead cells (10 min at 2000 × g) using a 5810 R centrifuge (Eppendorf). Cell debris were removed (30 min at 10,000 × g) and EVs were pelleted (70 min at 110,000 × g) using the Sw28 and the Sw32 Ti rotor (Beckman Coulter) in an L8–70 M machine (Beckmann). Preparations were extensively washed with PBS and pelleted (70 min at 110,000 × g) using a RP100-AT4 rotor (Sorvall) and a RC-M100 micro ultracentrifuge (Sorvall). All steps were performed at 4 °C and EVs were immediately processed for analysis.

### Electron microscopy

Steps were performed as previously described^89^. Briefly, 10 μL of fresh EVs preparations diluted in 100 μL PBS were loaded on previously discharged formvar-coated copper grids and allowed to adhere for 20 min. The grids were then applied to freshly prepared drops of 2% uranyl acetate for 30 to 40 s and washed six times for two minutes with water. Excessive water was removed by absorption and grids were left to dry for 30 min. Samples were imaged on a Tecnai 12 120 kV transmission electron microscope. Contrast was enhanced with the software Photoshop (Adobe).

### Nanoparticle Tracking Analysis

Cell depleted supernatants (30 min at 2000 × g) were analyzed by nanoparticle tracking analysis using an LM-10 machine (Nanosight) according to the manufacturer’s instructions. Samples were submitted to 3 successive analyses of 30 s using the default settings of the instrument. 3 washes were performed with water between samples. DMEM, M3 and Schneider media containing depleted FBS were used as negative controls. Biological triplicates were analyzed.

### Isolation and characterization of exRNA

EVs and corresponding cellular pellets were resuspended in 1 mL of TRIzol^TM^ reagent (Ambion) and processed according to the manufacturer’s instructions RNA extracts were purified with the RNA Clean & Concentrator^TM^-5 system (Zymo Research). *In-column* DNase I (New England BioLabs) treatment, RNA washes and elution steps were performed according to the manufacturer’s instructions. One additional centrifugation step was included (5 min at 16,000 × g) to ensure complete removal of the washing buffer. RNA samples were eluded in 12 μL of RNAse-free water (Wisent). Absorbance distributions were immediately quantified using a NanoDrop 2000c spectrophotometer. RNA samples were pure (A_260_/A_280_ ≥ 2.0)(2.00 ≤ A_260_/A_230_ ≤ 2.25). Aliquots of ~5 ng were submitted to capillary electrophoresis on a Bioanalyzer 2100 machine (Agilent). RNAse protection assay was performed as previously described^19^ to confirm the intraluminal topology of exRNA within EVs. D17 and HepG2 cellular RNA extracts were used as controls. RNAse A (Qiagen) was inactivated by heat (10 min at 65 °C) and RNA was extracted as described above. For calculations related to the estimation of RNA copy number per EV, we averaged the mass of each nucleoside at 325 Da. For example (1 ag of a 50 nt RNA): 10^−18^ g × 6.02 × 10^23^ molecules × mol^−1^ × (50 nt × molecule^−1^ × 325 g × mol^−1^ × molecule)^−1^ = 37.04 molecules.

### Library generation for RNA RNA-seq

Biological duplicates of sequencing libraries were prepared from high quality RNA extracts (50 ng exRNA and 500 ng cellular RNA) using the Illumina TruSeq Stranded RNA Kit according to the manufacturer’s instructions. The TruSeq PE Clusterkit v3-cBot-HS was used on an Illumina HiSEq 2000 machine.

### *In silico* analysis of RNA sequencing data

Read quality was confirmed using FastQC v0.10.1 and trimming was performed with Trimmomatic when deemed necessary. Read alignment was performed using Tophat v2.0.10 on the human GRCh37/hg19 and the *Drosophila* BDGP5.78/dm3 genomes, respectively. Alignment BAM files were used to generate bigWig files, which were submitted to the UCSC genome browser for read coverage visualization. Reads mapping to RepeatMasker v4.0.6 sequence coordinates were counted with BEDTools. Ribosomal RNA sequences were filtered out by first mapping the reads to FASTA files of genes annotated as rRNA in Ensembl. Remaining reads were then re-aligned to reference genomes with Tophat. BAM files were used for expression analyses with Cuffdiff v2.2.1 without effective length correction. Transcripts per million (TPM_i_ = [FPKM_i_/Σ_j_FPKM_j_] ×10^6^) were used as relative abundance units. A 5 FPKM threshold was applied. For biotype and correlation analyses, the 1,000 most abundant transcripts were considered, which accounted for over 90% of identified transcripts.

### Reverse transcription quantitative PCR (RT-qPCR)

The M-MLV reverse transcriptase (Invitrogen) was used according to the manufacturer’s instructions to synthesize cDNA in triplicate starting from 100 ng of exRNA and cell RNA. Priming was performed with random hexamers (Promega). Real-time PCR was performed on the ABI ViiA7 instrument (Life) using SYBR Green reagent (Applied Biosystems). Cycling conditions were as follows : 50 °C for two minutes and 95 °C for 10 min followed by 40 cycles of 95 °C for 15 s, 59 °C for 60 s. Melting curve analysis was performed. Validated primer sequences targeting exon junctions were retrieved from the GETprime database^86^ (Table 3).

### Functional annotation analysis

The 1,000 most abundant mRNAs in each EV type were used for functional analysis. Process, function and component GO terms were retrieved with DAVID. Terms associated with FDR < 10^−3^ were considered.

### General statistics

Statistic tests were performed with GraphPad Prism v6.

### Data access

Datasets are available on GEO under the accession number GSE76173.

## Additional Information

**How to cite this article**: Lefebvre, F. A. *et al*. Comparative transcriptomic analysis of human and *Drosophila* extracellular vesicles. *Sci. Rep.* **6**, 27680; doi: 10.1038/srep27680 (2016).

## Supplementary Material

Supplementary Information

## Figures and Tables

**Table 1:** Read metrics of human and *Drosophila* rRNA sequences.

Library	Mapped reads (×10^6^)	rRNA reads (×10^6^)	rRNA reads (%)
S2R + exRNA (i)	15.13	12.84	84.91
S2R + exRNA (ii)	19.29	17.74	91.98
D17 exRNA (i)	16.7	15.03	90.01
D17 exRNA (ii)	15.09	14.24	94.41
D17 cell RNA (i)	40.89	0.83	2.05
D17 cell RNA (ii)	38.01	0.86	2.27
A431 exRNA (i)	8.10	7.96	98.39
A431 exRNA (ii)	5.42	5.16	95.29
HepG2 exRNA (i)	7.06	6.45	91.38
HepG2 exRNA (ii)	6.97	5.55	79.73
HepG2 cell RNA (i)	71.51	0.49	0.69
HepG2 cell RNA (ii)	72.66	0.63	0.88

**Table 2:** Read metrics of human and *Drosophila* repeated elements and other transcripts.

Library	Reads left upon rRNA filtration (×10^6^)	Repeats reads (×10^6^)	Repeats reads (%)	Other RNA reads (×10^3^)	# Other RNA ≥ 5 FPKM
S2R + exRNA (i)	2.29	1.49	65.07	250	3,905
S2R + exRNA (ii)	1.55	1.09	70.32	180	
D17 exRNA (i)	1.67	0.83	49.70	81	4,472
D17 exRNA (ii)	0.85	0.74	87.06	51	
D17 cell RNA (i)	40.06	12.99	32.43	27,200	3,944
D17 cell RNA (ii)	37.15	11.83	31.84	25,300	
A431 exRNA (i)	0.14	0.02	16.43	110	9,969
A431 exRNA (ii)	0.26	0.05	18.85	270	
HepG2 exRNA (i)	0.61	0.10	16.39	850	4,754
HepG2 exRNA (ii)	1.42	0.24	16.90	2220	
HepG2 cell RNA (i)	71.02	4.92	6.93	68,850	6,537
HepG2 cell RNA (ii)	72.03	5.09	7.07	69,850	

**Table 3:** Primer sequences used for RT-qPCR validations.

*Homo sapiens* sequences		
	Forward primer	Reverse primer
*TPT1*	5′-CACGATGAGATGTTCTCCG-3′	5′-TCCTACTGACCATCTTCCC-3′
*PABPC1*	5′-CACTGGCATGTTGTTGGAG-3′	5′-CTTCATCAACCTTAGAACGGAG-3′
*ATF4*	5′-ATGATTACCTGGAGGTGGC-3′	5′-CTCCTTGCTGTTGTTGGAG-3′
*PTBP1*	5′-AGTTCTTCCAGAAGGACCG-3′	5′-GTTGTGCAGGTCAATGAGG-3′
*HDGF*	5′-GTGACGGTGATAAGAAGGG-3′	5′-TTTAGGAGAGTCCTCCAGC-3′
*G3BP1*	5′-GTAGAGGAACCTGAAGAAAGAC-3′	5′- ATGTCATTACTGACAACTGCC-3′
*BRAF*	5′- CTATTGGACAAATTTGGTGGG-3′	5′-GTATTCTTCATAGGCCTCCAG-3′
*Drosophila melanogaster* sequences		
	Forward primer	Reverse primer
*arc1*	5′-TAGAAGGTATCAGCGACGAG-3′	5′-GCCATACCGTAGAACAGCA-3′
*pabp2*	5′-GCATACATTGAGTTTGGTTCC-3′	5′-CGACATTACCTTTATTTGACGC-3′
*sqd*	5′-CACGGCAAGATCTTTGTCG-3′	5′-TCCACCTCGACGATATTGC-3′
*cam*	5′-GAAACTCACAGACGAGGAG-3′	5′-CATCATAGTCACGAATTCTTCG-3′
*rin*	5′-CAAGGGTGACTTTGAGCAG-3′	5′-GACATTTCCGAAGCGTGAG-3′
*hmu*	5′-TCTACTGGACGGACTCCTC-3′	5′-TATTTGAAGAGACGGCCGG-3′
*imp*	5′-CTCTACGAATAAGGGTGAACTC-3′	5′-CGTCCAATCAAATTGTTGTGG-3′
