# Supplementary material for: Comparative transcriptomic analysis of human and Drosophila extracellular vesicles
Source: Sci Rep. 2016 Jun 10;6:27680. doi: 10.1038/srep27680 (PMC4901365; doi:10.1038/srep27680)
Supplement: Supplementary Information [file srep27680-s1.pdf]

SUPPLEMENTAL INFORMATION

Comparative transcriptomic analysis of human and *Drosophila* extracellular vesicles

Fabio Alexis Lefebvre[1,2], Louis Philip Benoit Bouvrette[1,2], Lilyanne Perras[1,2], Alexis Blanchet-Cohen[1], Delphine Garnier[3,4], Janusz Rak[3], Éric Lécuyer[1,2,5,*]

1- Institut de Recherches Cliniques de Montréal (IRCM), Montréal, QC H2W 1R7, Canada
2- Département de Biochimie, Université de Montréal, Montréal, QC H3T 1J4, Canada
3- Montreal Children's Hospital, Research Institute of the McGill University Health Centre, Montreal, QC H4A 3J1, Canada
4- INSERM UMR 1064-ITUN; CHU de Nantes, Nantes 44093, France
5- Division of Experimental Medicine, McGill University, Montréal, QC H3A 1A3, Canada

***-** Corresponding author:

Dr. Eric Lécuyer
IRCM, RNA Biology Laboratory
110 Avenue des Pins, Ouest
Montréal, Québec, Canada, H2W 1R7
Email: eric.lecuyer@ircm.qc.ca

Supplemental Figure Legends

Figure S1. EV count per preparation. EV counts were determined on the basis of NTA analysis, considering total particles in cultures established from 10^7 cells over the course of 48h. The significance of the differences observed between lines of the same species was evaluated through a Mann-Whitney test, while the prevalence of a global difference was evaluated using a Kruskal-Wallis test. Differences are deemen non-significant.

Figure S2. Human and *Drosophila* EVs contain similar amounts of proteins and RNA. Quantification of total protein (**left panel**) and RNA (**right panel**) in EV preparations derived from human and *Drosophila* cells. No significant difference was found across samples by an ANOVA test.

Figure S3. Average protein and RNA content per individual EV. Estimations of total protein (top panel) and RNA (bottom panel) mass found in individual EV were obtained from results of photometric macromolecule quantification and NTA vesicle counts. The significance of the differences observed between lines of the same species was evaluated through a Mann-Whitney test while the prevalence of a global difference was evaluated using a Kruskal-Wallis test. One femtogram (fg) corresponds to 10^{-15} g, while one attogram (ag) corresponds to 10^{-18} g.

Figure S4. The bulk of RNA found in human and *Drosophila* EVs is protected from degradation by RNAse A. EV preparations derived from human HepG2 and A431 and *Drosophila* D17 and S2R+ cells were split in two equal pools. The first pool (grey bars) was incubated with RNAse A followed by inactivation and total RNA extraction, while the second pool (black bars) was directly submitted to RNA extraction. Previously quantified samples of cellular HepG2 and D17 total RNA were used as controls.

Figure S5. RNA in human and *Drosophila* EVs largely consists of short sequences. Length distributions of human and *Drosophila* EV (**left panels**) and cellular (**right panels**) total RNA were evaluated by bioanalyzer capillary electrophoresis. 'R' refers to the 18S and 28S rRNA peaks, while 'M' refers to the exogenous marker peak.

Figure S6. Bidirectional read distributions map to 5S pseudogenes in human HepG2 EVs. Strand-specific read coverage of the genomic region surrounding the *RNA5SP145* (chr3:150,905,886-150,906,010, **top panel**) and *RNA5SP149* (chr3:179,879,674-179,879,765; **bottom panel**) in human HepG2 EVs and cells. The *y*-axis refers to read coverage (no normalization). Screen shots were taken from the UCSC genome browser.

Figure S7. Mitochondrial rRNAs display abundant bidirectional read coverage in HepG2 EVs and cells. Strand-specific read coverage of the genomic region surrounding the 12S (*MT-RNR1*; chrM:650-1,603) and 16S (*MT-RNR2*; chrM:1,673-3,230) loci in human HepG2 EVs and cells. Note that rRNA depletion was performed prior to RNA-seq in HepG2 cellular extracts.

Figure S8. Abundant bidirectional signatures of an rRNA-like novel miRNA in human HepG2 EVs. Strand-specific read coverage of the genomic region surrounding the *AC079949.1*

“novel miRNA” locus in human HepG2 EVs and cells. This miRNA overlaps with a large subunit rRNA repeat (chr12:127,650,453-127,650,987), H3K27 acetylated and DNAse I sensitive region.

Figure S9. Abundant full-length read distributions map to vault RNA 1-1 in HepG2 EVs. Strand-specific read coverage surrounding the *VTRNA1-1* locus (chr5:140,090,861-140,090,958) in HepG2 EVs and cells.

Figure S10. Spliceosomal U5 snRNAs are abundant in human and *Drosophila* EVs. Strand-specific read coverage of a *Drosophila* U5 snRNA gene (*U5:63BC*; chr3L:3,090,801-3,090,923) and a human ortholog (*RNU5B-1*; chr15:65,597,015-65,597,130) in D17 (**A**) and HepG2 (**B**) EVs and cells.

Figure S11. Intronic snoRNAs display differential targeting to *Drosophila* EVs. Strand-specific read coverage of three snoRNAs encoded from intronic regions of *CG13900* (chr3L:826,720-833,385) in *Drosophila* EVs and cells.

Figure S12. Evidence of tRNA targeting to *Drosophila* and human EVs. Strand-specific read coverage of two proximal *Drosophila* tRNAs (chr2L:8,683,381-8,683,935; **top panel**) and two human tRNAs (chr12:125,423,890-125,425,085; **bottom panel**). The latter example shows evidence of bidirectional tRNA sequences in EVs and asymmetric read coverage in EVs and cells.

Figure S13. A narrow read distribution maps to a central region of the retrotransposon *TART* in *Drosophila* EVs and cells. Strand-specific read coverage of the *TART* locus (chrX:15,069-25,164) in *Drosophila* EVs and cells.

Figure S14. A narrow antisense read distribution maps to a central region of the retrotransposon *Copia* in *Drosophila* EVs. Strand-specific read coverage surrounding a *Copia* element repeat (chr2L:2,294,373-2,298,967) in *Drosophila* S2R+ EVs, D17 EVs and D17 cells.

Figure S15. Correlative analysis of orthologous protein-coding genes. Relative TPM abundance values of *Drosophila* D17 mRNAs were correlated to their corresponding human HepG2 orthologs. Cellular (**top panel**) and EV (**bottom panel**) populations are displayed as scatter plots. Pearson’s correlations (r) and associated p-values are indicated at the top of each graph.

Supplemental Table Legends

Table S1. Read distributions of the fifty most abundant rRNA genes in human and *Drosophila* EV samples.

Table S2. List of the ten most abundant transcripts in human and *Drosophila* EV samples following rRNA subtraction.

Table S3. List of the ten most abundant repeat element-derived RNAs in human and *Drosophila* EVs.

Table S4. List of the ten most abundant mRNAs in human and *Drosophila* EVs.

Table S5. List of the ten most significantly enriched gene ontology (GO) terms associated with human and *Drosophila* EV mRNAs.

Table S6. Exhaustive list of "EV-exclusive" mRNAs in human HepG2 and *Drosophila* D17 cells.

Figure S1

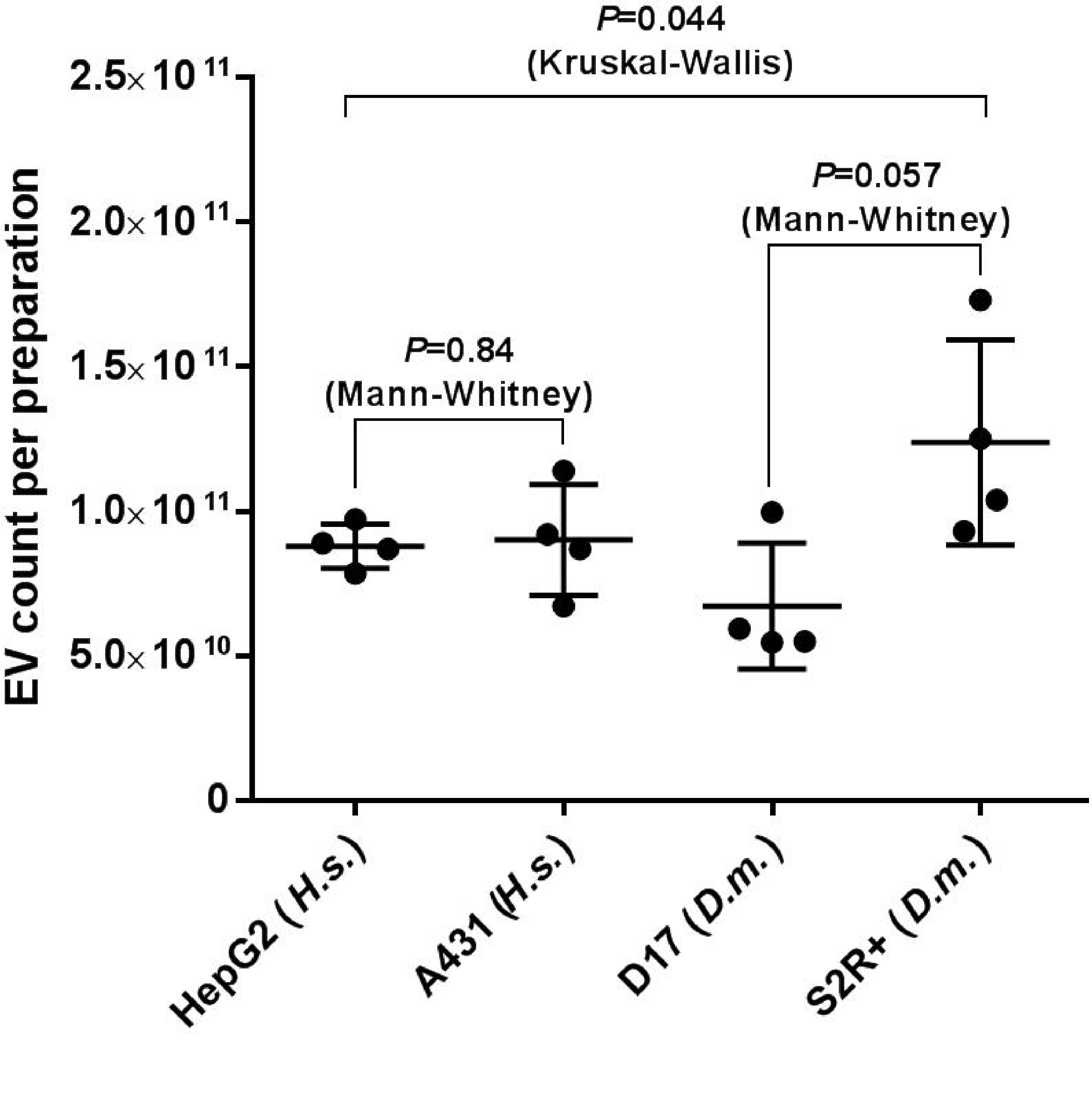

P=0.044
(Kruskal-Wallis)
P=0.057
(Mann-Whitney)
P=0.84
(Mann-Whitney)
EV count per preparation
2.5×10 11
2.0×10 11
1.5×10 11
1.0×10 11
5.0×10 10
0
HepG2 (H.s.)
A431 (H.s.)
D17 (D.m.)
S2R+ (D.m.)

Figure S2

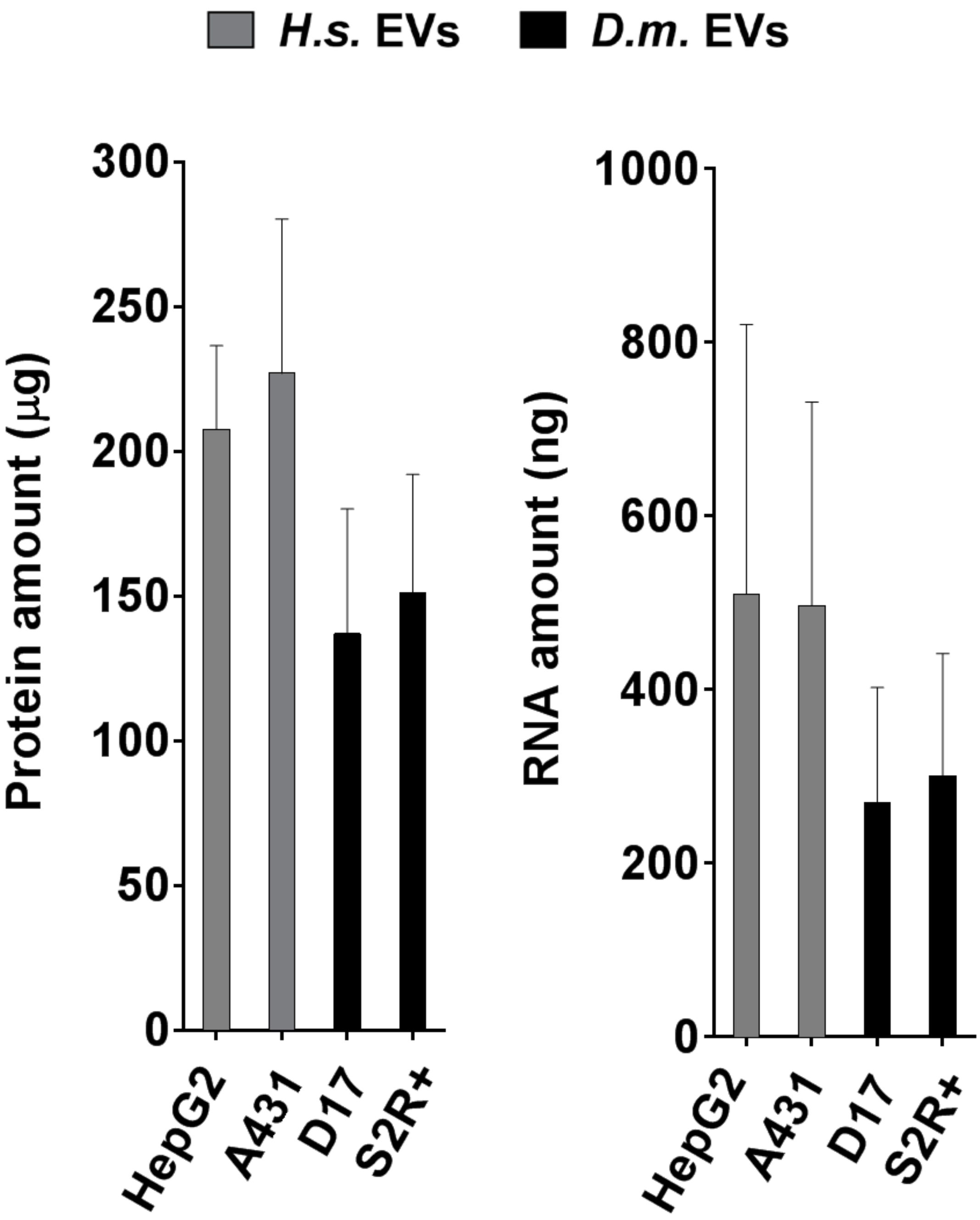

H.s. EVs
D.m. EVs
Protein amount (μg)
300
250
200
150
100
50
0
HepG2
A431
D17
S2R+
RNA amount (ng)
1000
800
600
400
200
0
HepG2
A431
D17
S2R+

Figure S3

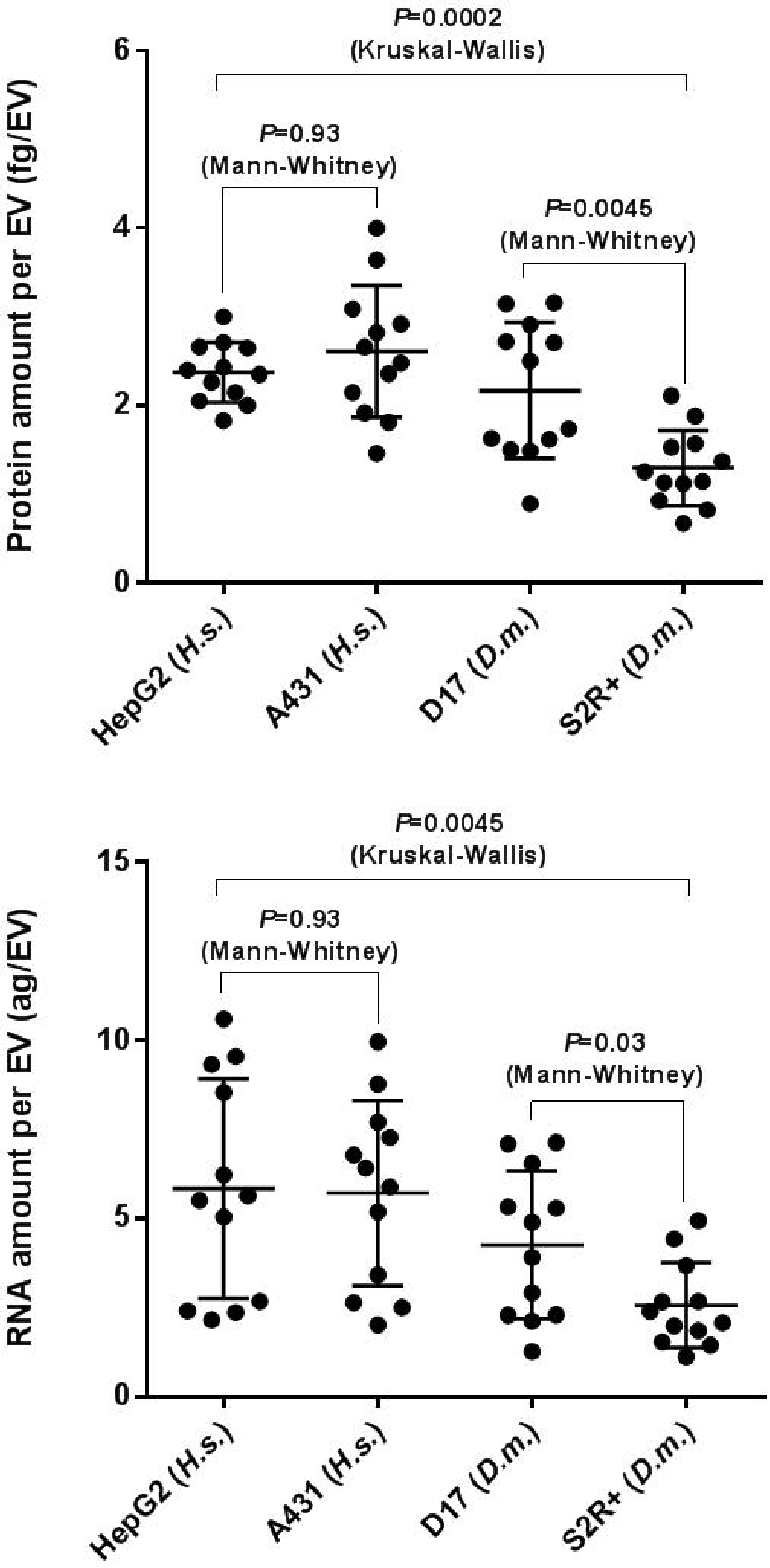

P=0.0002
(Kruskal-Wallis)
P=0.93
(Mann-Whitney)
P=0.0045
(Mann-Whitney)
Protein amount per EV (fg/EV)
6
4
2
0
HepG2 (H.s.)
A431 (H.s.)
D17 (D.m.)
S2R+ (D.m.)
P=0.0045
(Kruskal-Wallis)
P=0.93
(Mann-Whitney)
P=0.03
(Mann-Whitney)
RNA amount per EV (ag/EV)
15
10
5
0
HepG2 (H.s.)
A431 (H.s.)
D17 (D.m.)
S2R+ (D.m.)

Figure S4

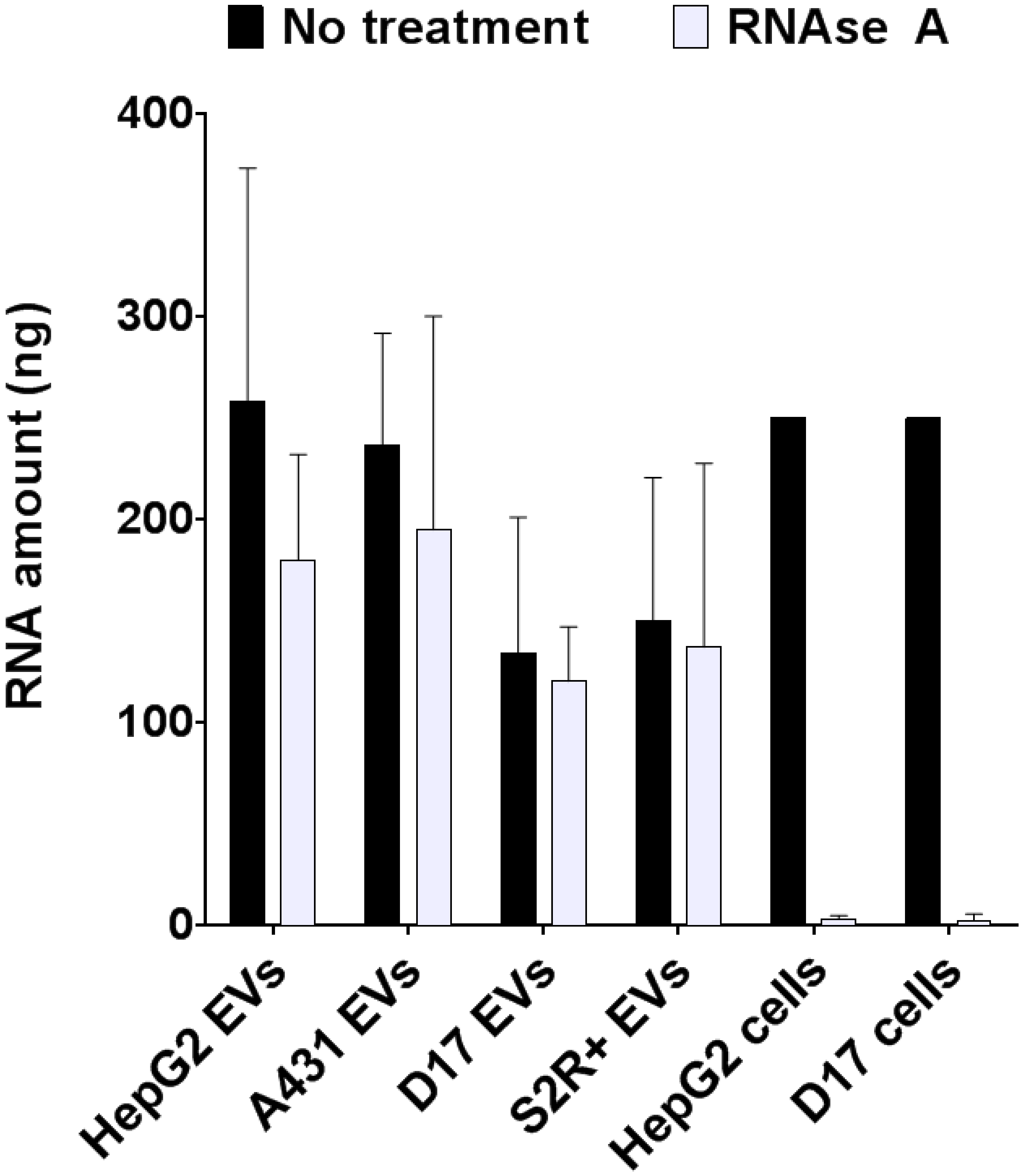

No treatment
RNAse A
RNA amount (ng)
400
300
200
100
0
HepG2 EVs
A431 EVs
D17 EVs
S2R+ EVs
HepG2 cells
D17 cells

Figure S5

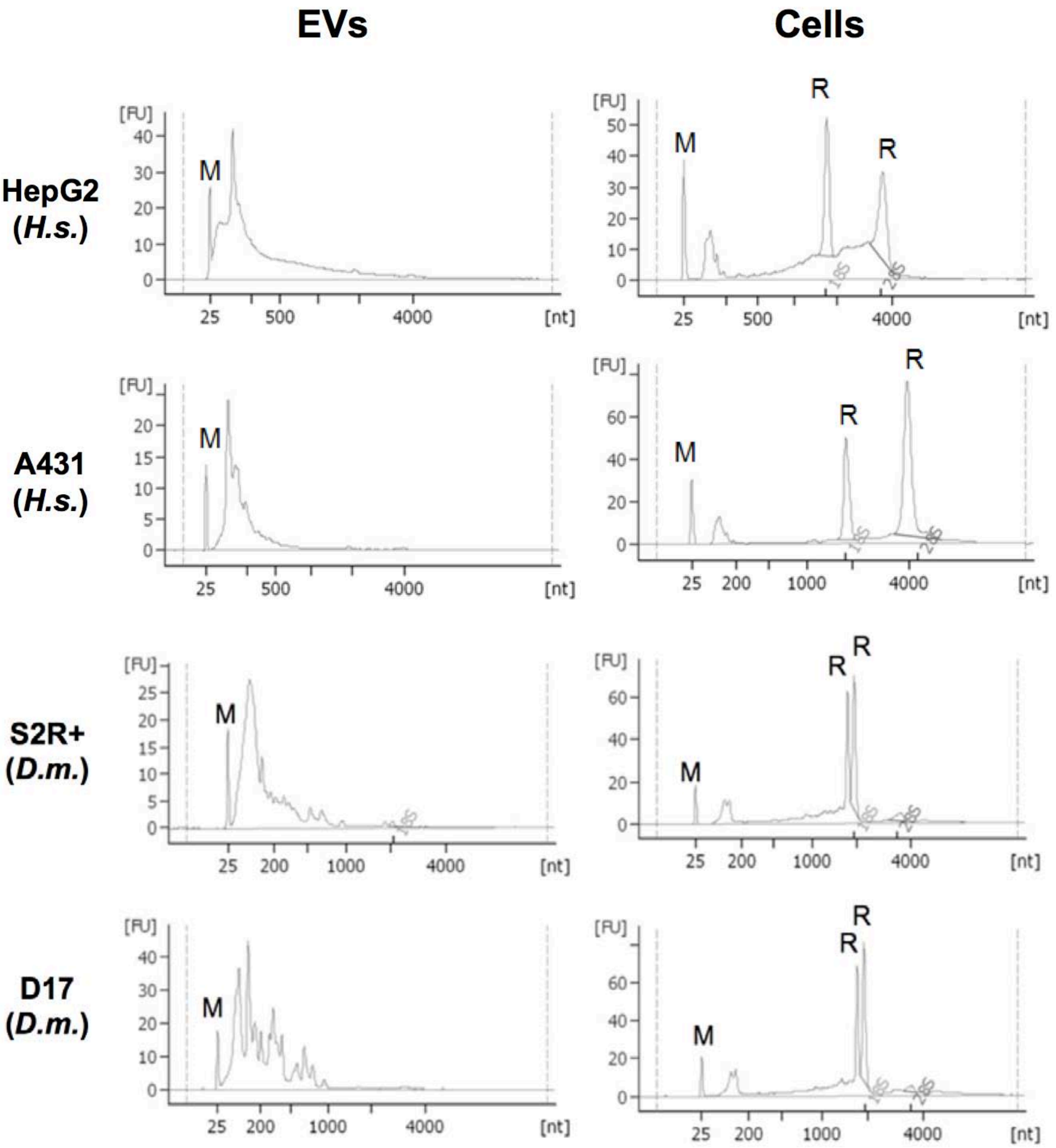

EVs
Cells
HepG2
(*H.s.*)
A431
(*H.s.*)
S2R+
(*D.m.*)
D17
(*D.m.*)
M
R
[FU]
[nt]
25
200
500
1000
4000
18S
28S

Figure S6

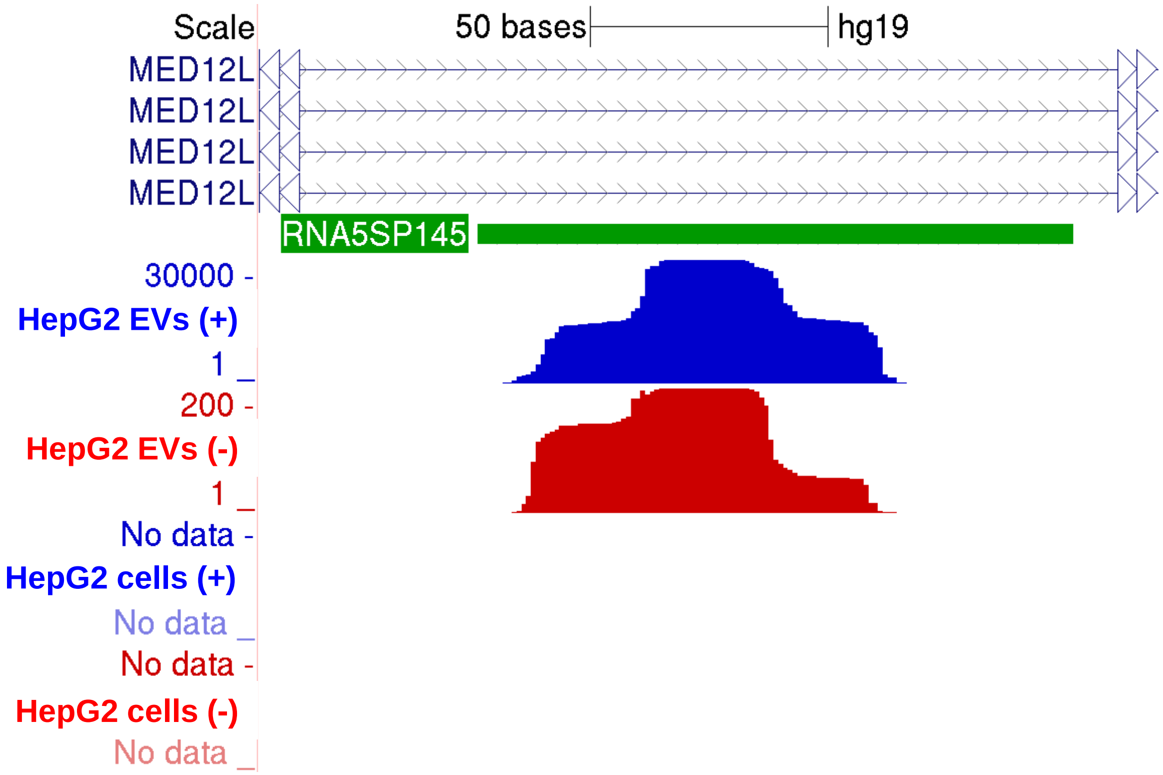

Scale
50 bases
hg19
MED12L
MED12L
MED12L
MED12L
RNA5SP145
30000 -
HepG2 EVs (+)
1 _
200 -
HepG2 EVs (-)
1 _
No data -
HepG2 cells (+)
No data _
No data -
HepG2 cells (-)
No data _

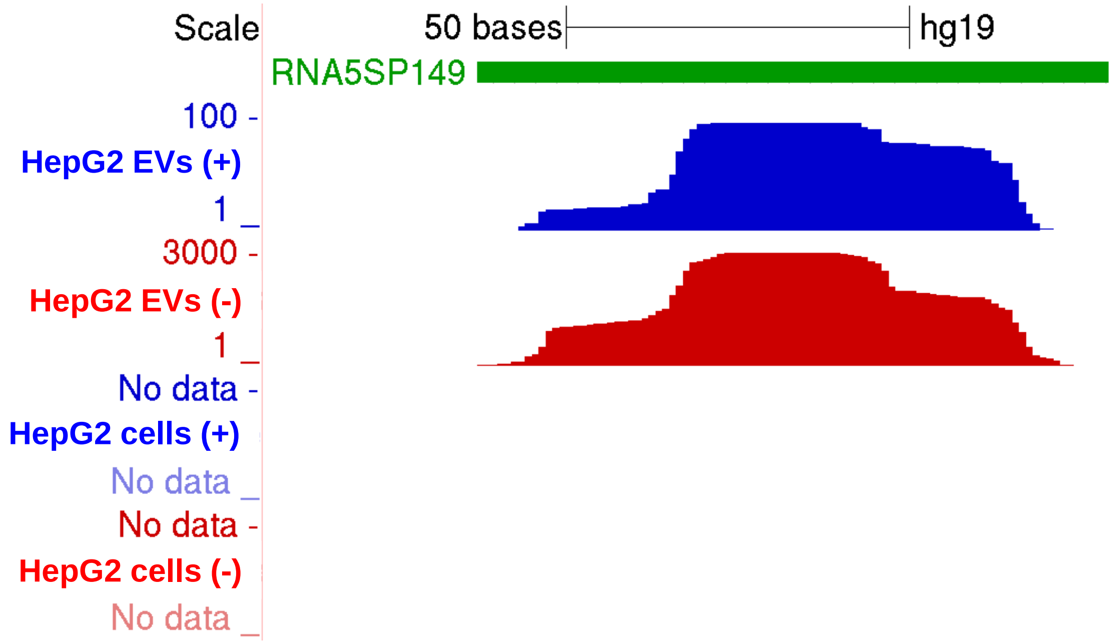

Scale
50 bases
hg19
RNA5SP149
100 -
HepG2 EVs (+)
1 _
3000 -
HepG2 EVs (-)
1 _
No data -
HepG2 cells (+)
No data _
No data -
HepG2 cells (-)
No data _

Figure S7

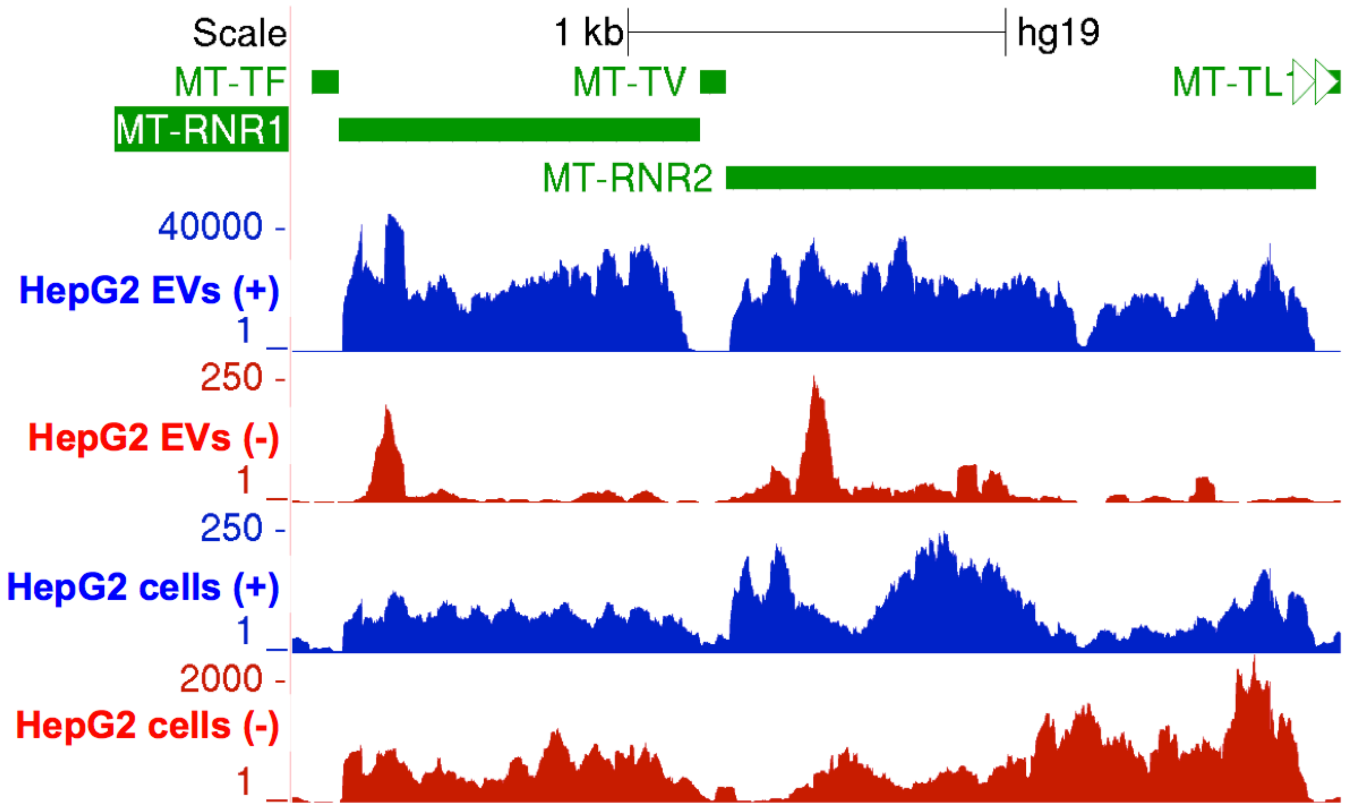
Scale
1 kb
hg19
MT-TF
MT-TV
MT-TL1
MT-RNR1
MT-RNR2
40000 -
HepG2 EVs (+)
1 _
250 -
HepG2 EVs (-)
1 _
250 -
HepG2 cells (+)
1 _
2000 -
HepG2 cells (-)
1 _

Figure S8

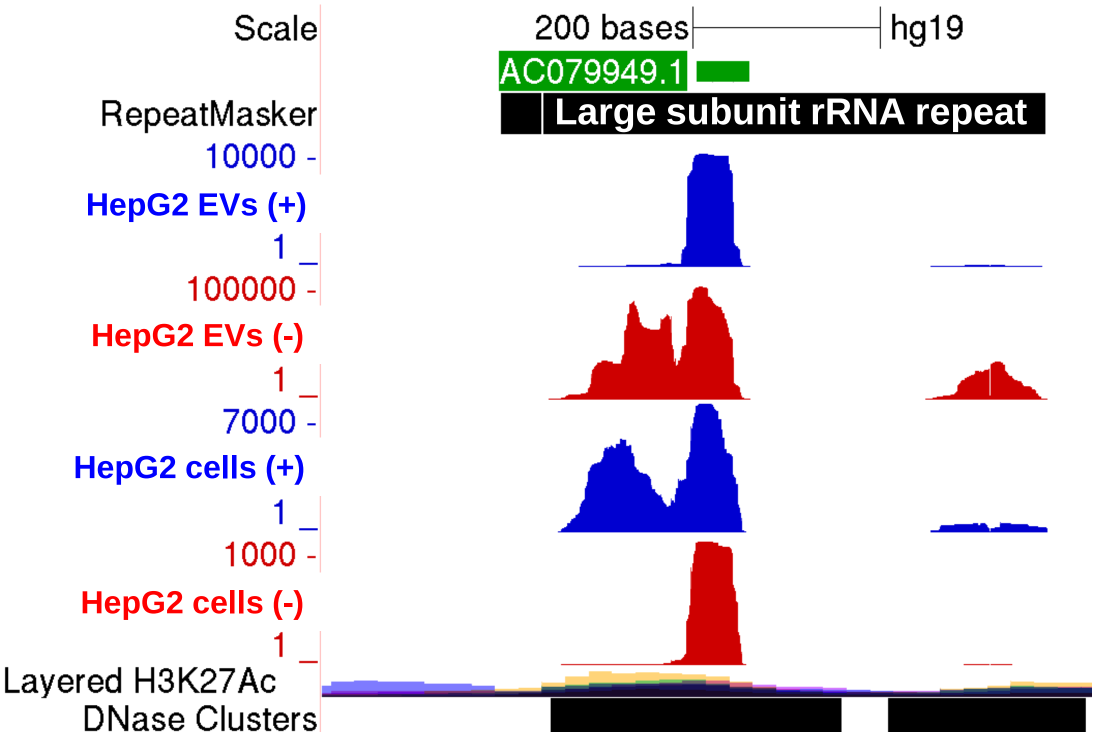

Scale
200 bases
hg19
AC079949.1
RepeatMasker
Large subunit rRNA repeat
10000 -
HepG2 EVs (+)
1 _
100000 -
HepG2 EVs (-)
1 _
7000 -
HepG2 cells (+)
1 _
1000 -
HepG2 cells (-)
1 _
Layered H3K27Ac
DNase Clusters

Figure S9

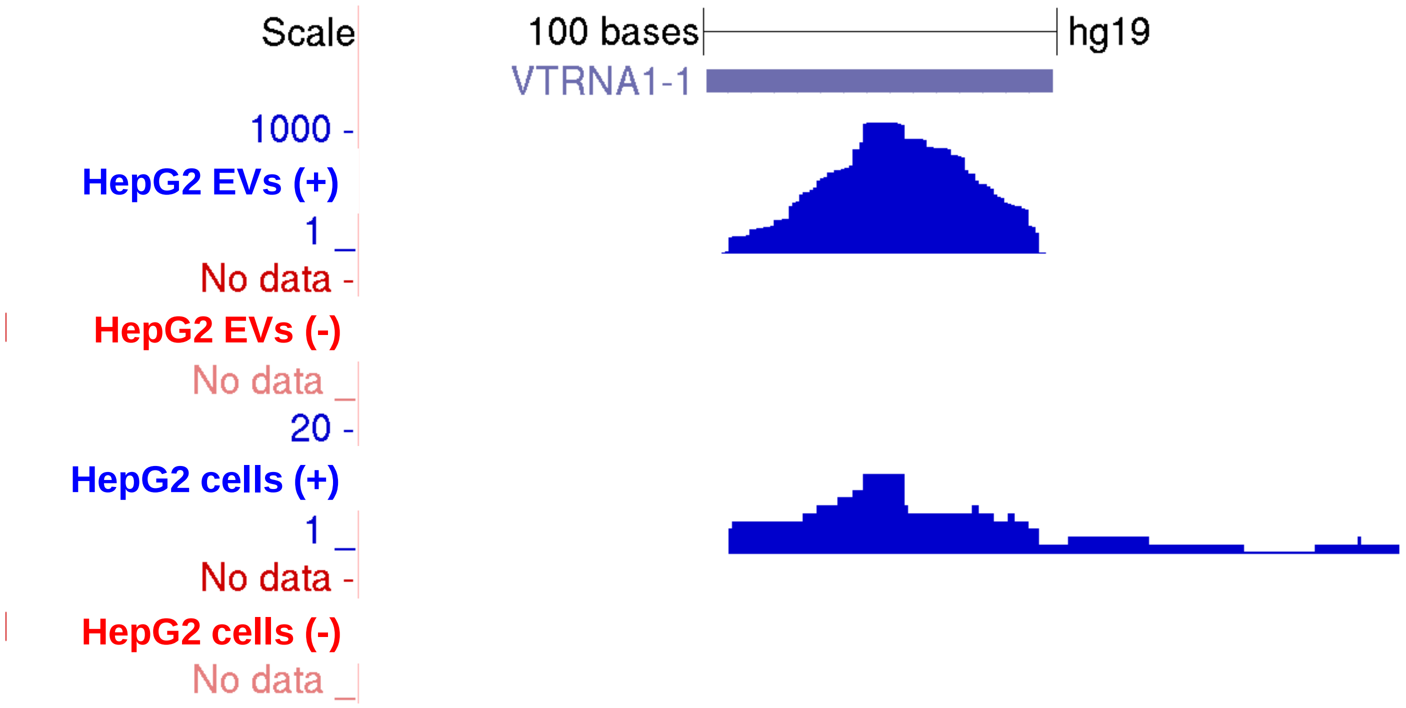
Scale
100 bases
hg19
VTRNA1-1
1000 -
HepG2 EVs (+)
1 _
No data -
HepG2 EVs (-)
No data _
20 -
HepG2 cells (+)
1 _
No data -
HepG2 cells (-)
No data _

Figure S10

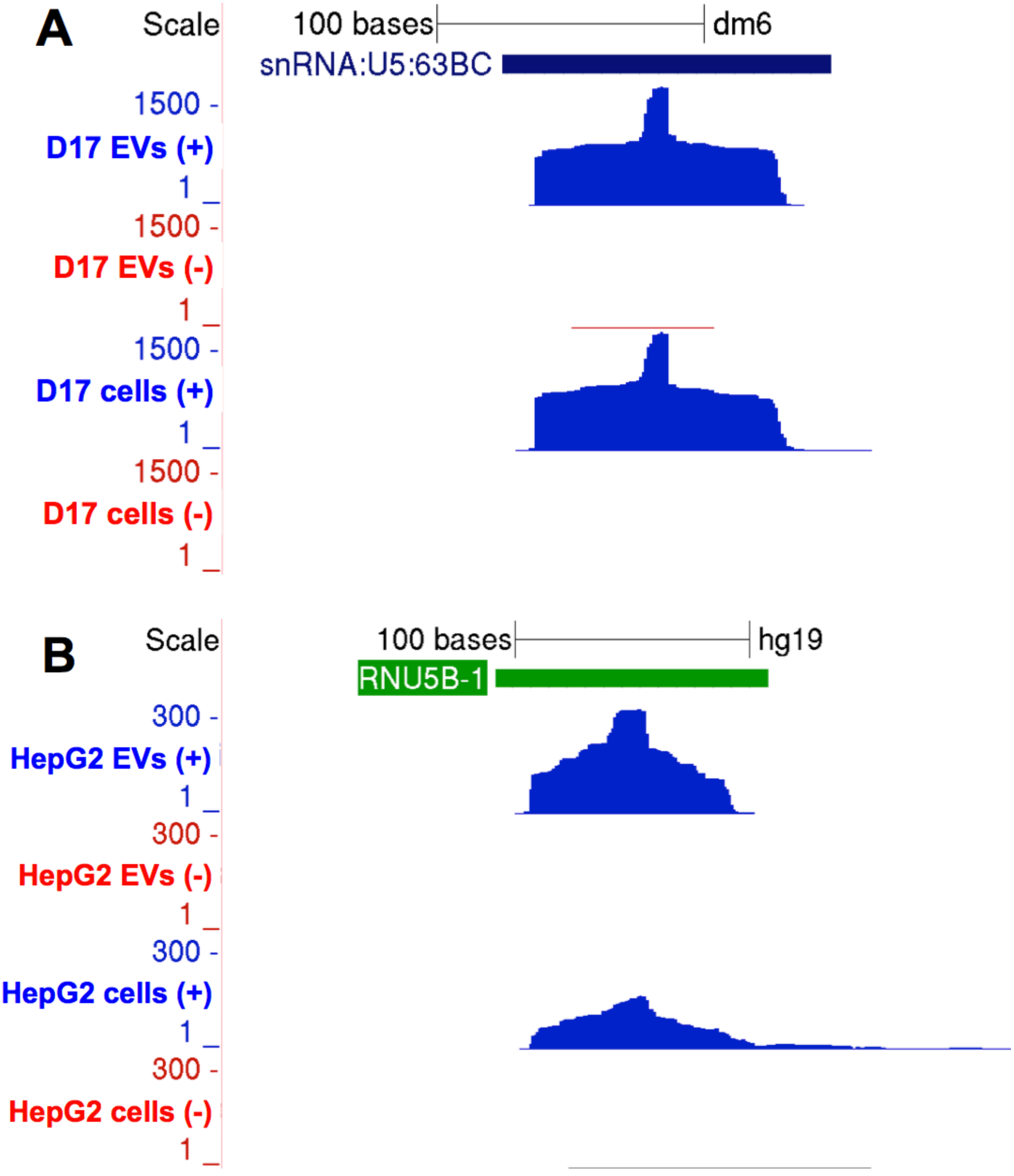
A
Scale
100 bases
dm6
snRNA:U5:63BC
1500 -
D17 EVs (+)
1 _
1500 -
D17 EVs (-)
1 _
1500 -
D17 cells (+)
1 _
1500 -
D17 cells (-)
1 _
B
Scale
100 bases
hg19
RNU5B-1
300 -
HepG2 EVs (+)
1 _
300 -
HepG2 EVs (-)
1 _
300 -
HepG2 cells (+)
1 _
300 -
HepG2 cells (-)
1 _

Figure S11

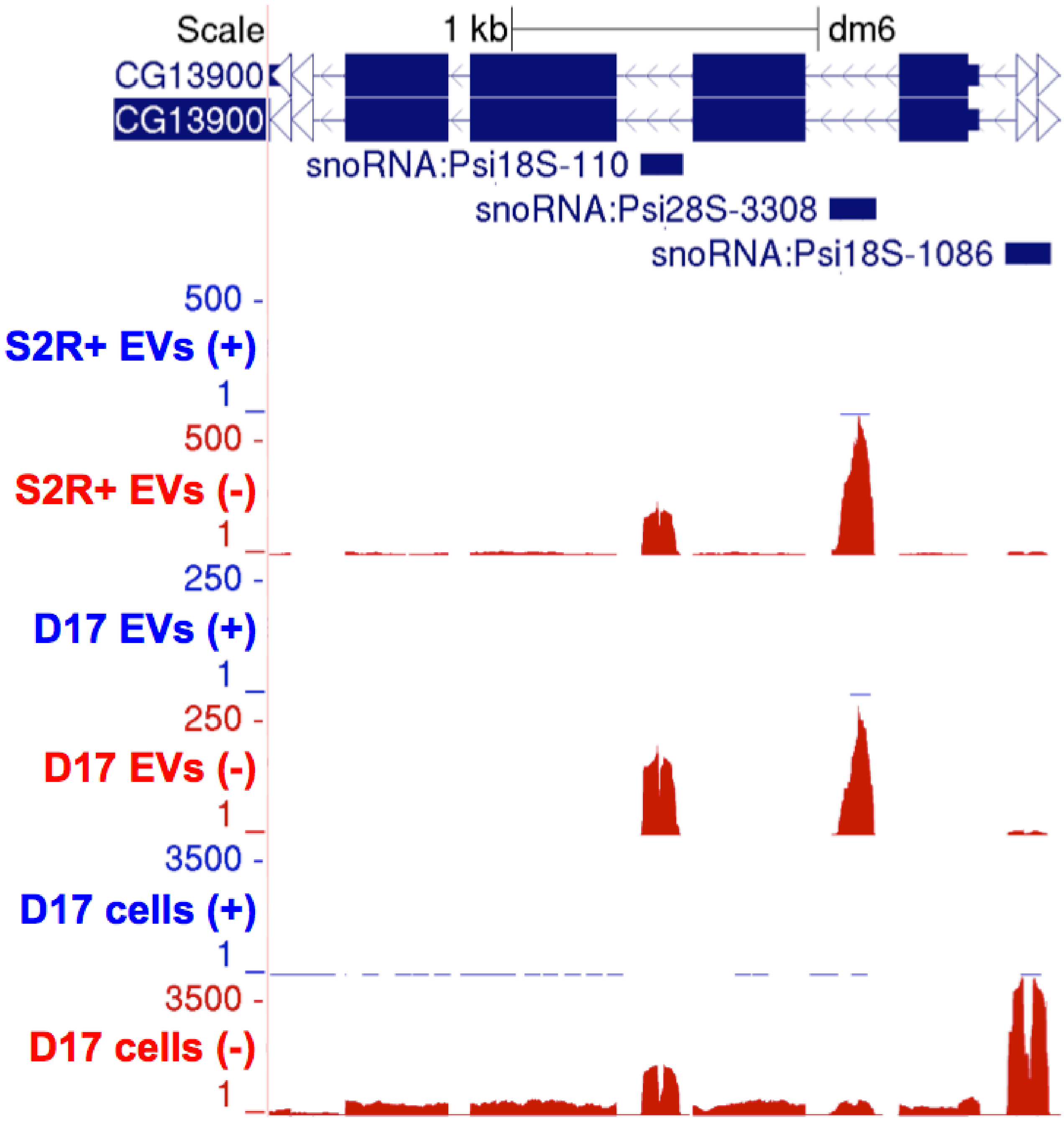

Scale
1 kb
dm6
CG13900
CG13900
snoRNA:Psi18S-110
snoRNA:Psi28S-3308
snoRNA:Psi18S-1086
500 -
S2R+ EVs (+)
1 _
500 -
S2R+ EVs (-)
1 _
250 -
D17 EVs (+)
1 _
250 -
D17 EVs (-)
1 _
3500 -
D17 cells (+)
1 _
3500 -
D17 cells (-)
1 _

Figure S12

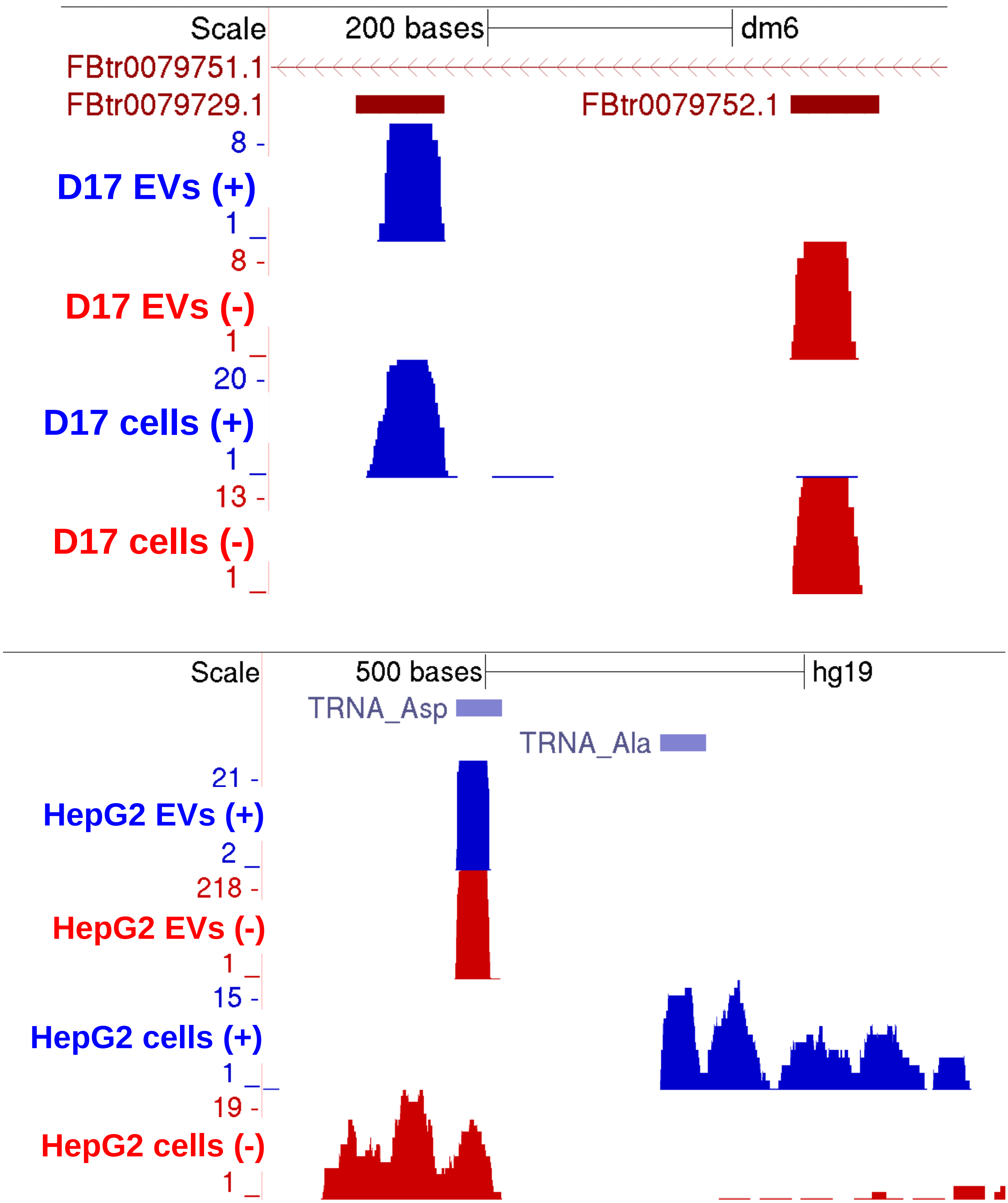
Scale
200 bases
dm6
FBtr0079751.1
FBtr0079729.1
FBtr0079752.1
8 -
D17 EVs (+)
1 _
8 -
D17 EVs (-)
1 _
20 -
D17 cells (+)
1 _
13 -
D17 cells (-)
1 _
Scale
500 bases
hg19
TRNA_Asp
TRNA_Ala
21 -
HepG2 EVs (+)
2 _
218 -
HepG2 EVs (-)
1 _
15 -
HepG2 cells (+)
1 _
19 -
HepG2 cells (-)
1 _

Figure S13

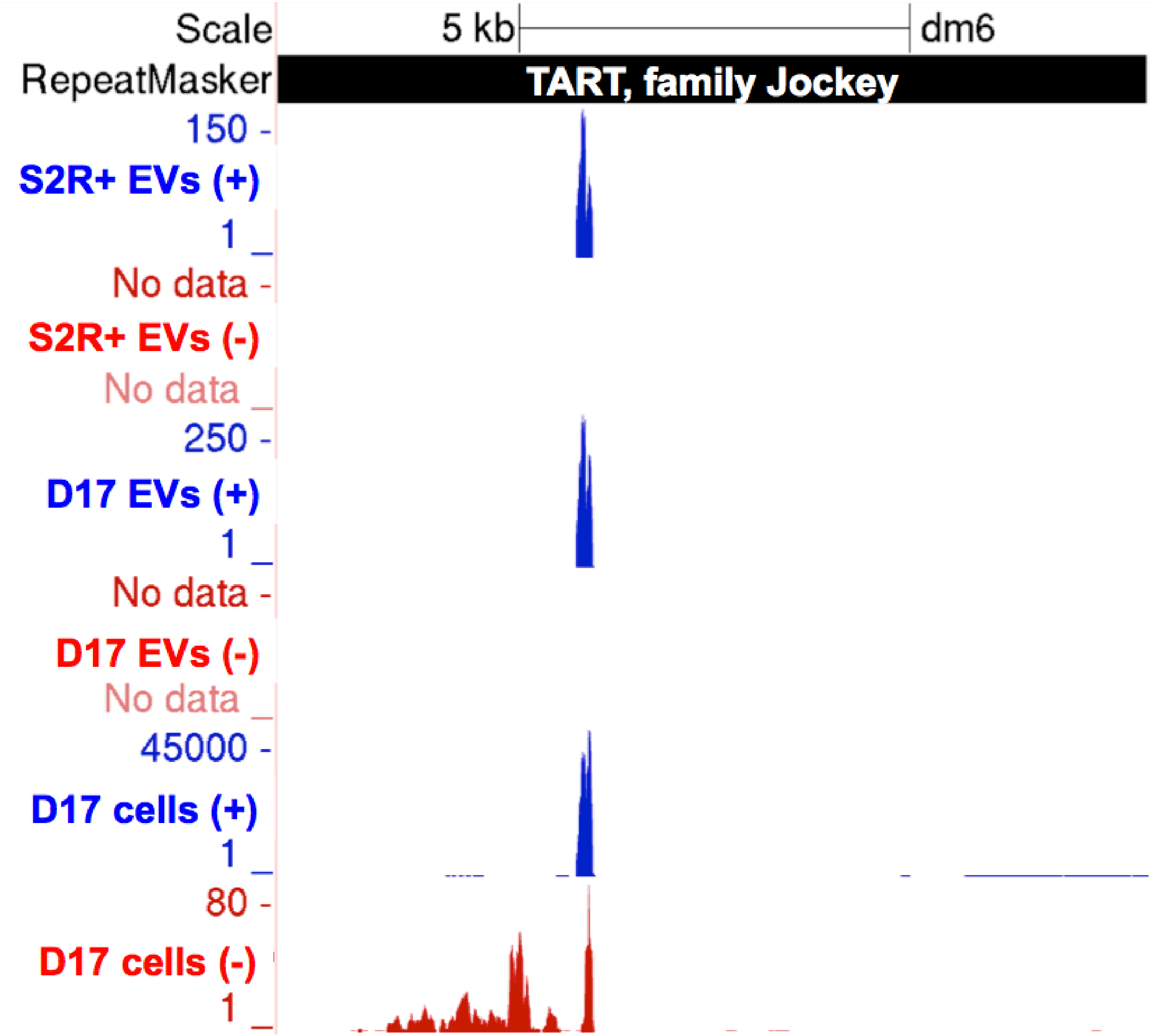
Scale
5 kb
dm6
RepeatMasker
TART, family Jockey
150 -
S2R+ EVs (+)
1 _
No data -
S2R+ EVs (-)
No data _
250 -
D17 EVs (+)
1 _
No data -
D17 EVs (-)
No data _
45000 -
D17 cells (+)
1 _
80 -
D17 cells (-)
1 _

Figure S14

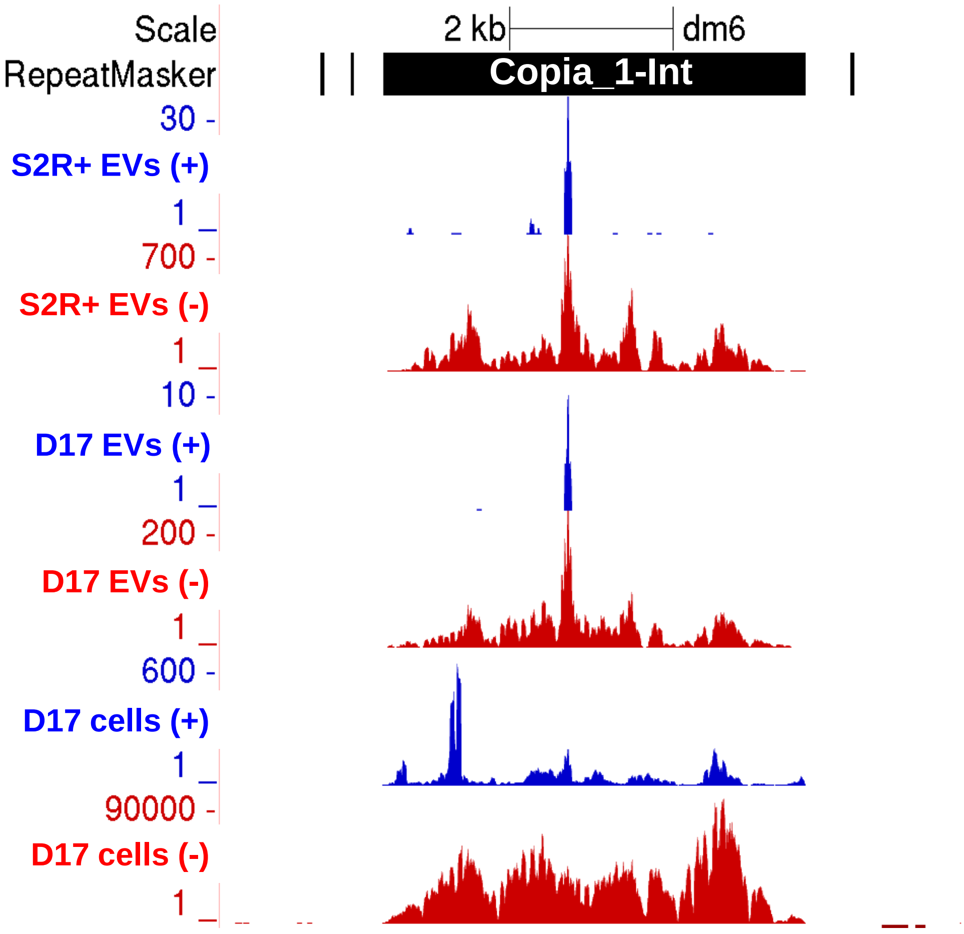
Scale
2 kb
dm6
RepeatMasker
Copia_1-Int
30 -
S2R+ EVs (+)
1 _
700 -
S2R+ EVs (-)
1 _
10 -
D17 EVs (+)
1 _
200 -
D17 EVs (-)
1 _
600 -
D17 cells (+)
1 _
90000 -
D17 cells (-)
1 _

Figure S15

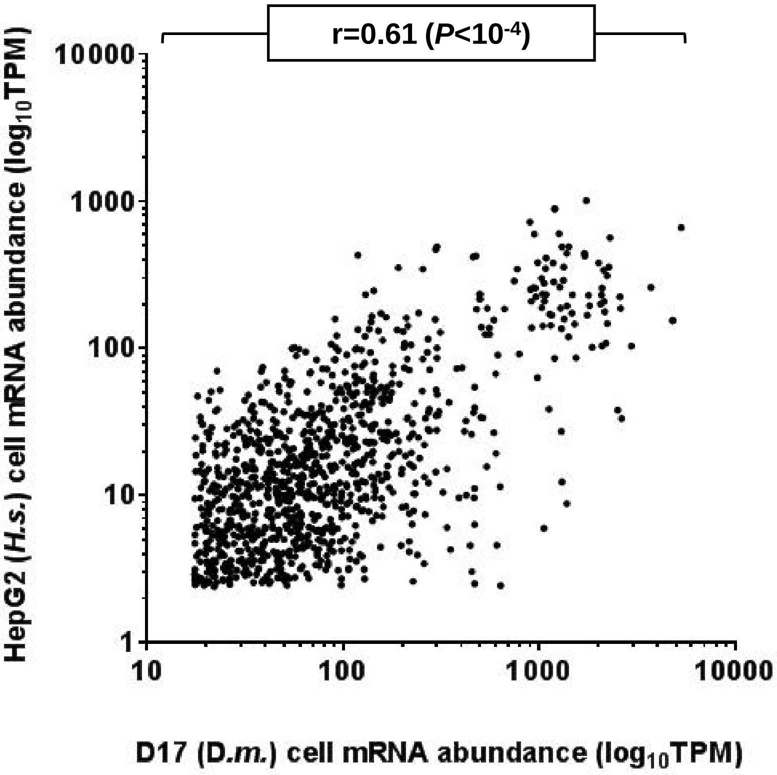

r=0.61 (P<10-4)
HepG2 (H.s.) cell mRNA abundance (log10TPM)
10000
1000
100
10
1
10
100
1000
10000
D17 (D.m.) cell mRNA abundance (log10TPM)

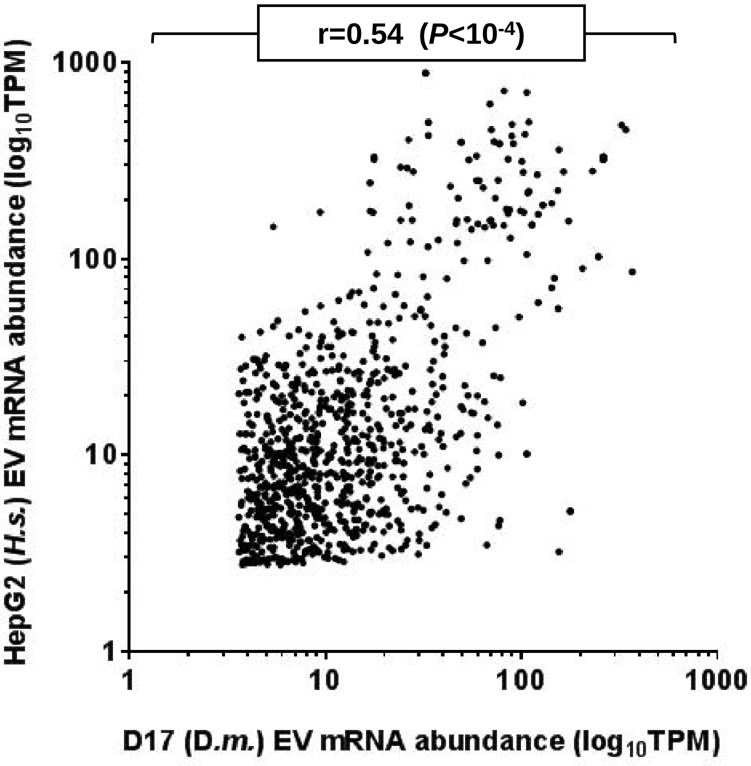

r=0.54 (P<10-4)
HepG2 (H.s.) EV mRNA abundance (log10TPM)
1000
100
10
1
1
10
100
1000
D17 (D.m.) EV mRNA abundance (log10TPM)

Table S1

ensembl_gene_id	gene_id	A431 Evs	HepG2 Evs	HepG2 cells
ENSG00000266658	RNA28S5	18320021	16414483	853851
ENSG00000272060	RNA18S5	15308624	10290624	285306
ENSG00000210082	MT-RNR2	81302	396901	26866
ENSG00000211459	MT-RNR1	29807	276904	12367
ENSG00000241335	RNA5-8S5	77325	66110	138
ENSG00000242716	RNA5-8S5	77305	66095	191
ENSG00000201925	RNA5S15	10704	23295	4
ENSG00000200624	RNA5S6	10313	23071	1
ENSG00000199910	RNA5S10	10265	22870	1
ENSG00000199352	RNA5S1	10554	23056	1
ENSG00000199337	RNA5S3	10362	22704	0
ENSG00000202257	RNA5S16	10289	22646	1
ENSG00000199334	RNA5S11	10052	22313	0
ENSG00000199270	RNA5S12	10487	22525	2
ENSG00000201588	RNA5S2	10323	22291	5
ENSG00000202526	RNA5S13	10111	22266	4
ENSG00000200343	RNA5S8	10221	22204	2
ENSG00000202521	RNA5S7	10236	22213	3
ENSG00000201355	RNA5S14	10259	22273	0
ENSG00000199396	RNA5S5	9988	21973	2
ENSG00000200381	RNA5S4	10216	21964	4
ENSG00000200370	RNA5S17	10105	21766	1
ENSG00000201321	RNA5S9	3951	5204	0
ENSG00000199415	RNA5SP370	2059	4456	0
ENSG00000199523	RNA5SP226	2045	4254	0
ENSG00000199480	RNA5SP357	1910	4096	0
ENSG00000199654	RNA5SP389	1917	4070	0
ENSG00000201185	RNA5SP202	2002	3199	0
ENSG00000199994	RNA5SP145	630	1380	0
ENSG00000201822	RNA5SP149	262	475	0
ENSG00000200741	RNA5SP161	332	344	0
ENSG00000252623	RNA5SP481	165	278	0
ENSG00000201861	RNA5SP298	132	255	0
ENSG00000222806	RNA5SP225	46	91	0
ENSG00000266658	RNA28S5	624	125	3
ENSG00000201413	RNA5SP141	18	45	0
ENSG00000200558	RNA5SP429	25	45	0
ENSG00000202411	RNA5SP259	21	34	0
ENSG00000199564	RNA5SP502	10	26	0
ENSG00000200278	RNA5SP352	16	25	0
ENSG00000251705	RNA5-8SP6	62	35	0
ENSG00000272351	5S_rRNA	10	9	0
ENSG00000199839	RNA5SP150	6	8	0
ENSG00000200275	RNA5SP199	7	8	0
ENSG00000201041	RNA5SP242	4	5	0
ENSG00000201096	RNA5SP387	2	4	0
ENSG00000200408	RNA5SP74	1	3	0
ENSG00000200687	RNA5SP335	7	4	0
ENSG00000202187	RNA5SP355	5	5	0
ENSG00000200115	RNA5SP440	0	2	0

Flybase ID	Gene name	S2R+ Evs	D17 Evs	D17 cell
FBgn0085797	CR41540	5306258	3591189	295662
FBgn0085813	CR41602	3517800	3380805	118729
FBgn0085742	CR40546	2429095	1567232	186204
FBgn0085774	CR40779	2260038	2136917	16865
FBgn0085805	CR41583	2106021	2505405	137649
FBgn0085823	CR41617	2092710	3075441	2922
FBgn0085819	CR41609	1596444	930954	134872
FBgn0085773	CR40766	1407175	726834	26546
FBgn0085825	CR41619	1344789	763627	4271
FBgn0085807	CR41590	868201	1204822	12725
FBgn0085765	CR40677	780867	816985	77306
FBgn0085815	CR41605	746258	1407179	6373
FBgn0085757	CR40621	588246	818844	10962
FBgn0085743	CR40560	560630	423825	27134
FBgn0085769	CR40728	472500	405368	1632
FBgn0085817	CR41607	423919	638905	3677
FBgn0085764	CR40668	405530	545564	148161
FBgn0085796	CR41539	379780	396090	4901
FBgn0085822	CR41613	376405	400427	3161
FBgn0085795	CR41535	375645	318151	62542
FBgn0085761	CR40642	372968	395060	3086
FBgn0085759	CR40640	287741	269986	2839
FBgn0085770	CR40734	263840	335867	56804
FBgn0085760	CR40641	255087	304084	2777
FBgn0085828	CR42195	231387	328168	3164
FBgn0085777	CR40959	230728	256935	937
FBgn0085799	CR41544	194809	250138	2754
FBgn0085771	CR40741	190510	545404	115922
FBgn0085766	CR40679	164617	202766	11915
FBgn0085753	CR40596	160787	144314	56757
FBgn0085758	CR40639	118711	82604	3938
FBgn0085802	CR41548	100029	100012	13876
FBgn0085737	CR40502	39134	87148	402
FBgn0085767	CR40685	23564	36117	22197
FBgn0085772	CR40743	23503	35637	1074
FBgn0250731	5.8SrRNA:CR40454	23359	35803	44
FBgn0085826	CR41620	23276	35703	388
FBgn0085827	CR41621	5352	9367	7148
FBgn0053394	5SrRNA:CR33394	2638	3443	60
FBgn0053435	5SrRNA:CR33435	2606	3258	54
FBgn0053358	5SrRNA:CR33358	2595	3264	66
FBgn0053388	5SrRNA:CR33388	2595	3230	62
FBgn0053426	5SrRNA:CR33426	2557	3183	67
FBgn0053373	5SrRNA:CR33373	2556	3189	64
FBgn0053425	5SrRNA:CR33425	2554	3281	60
FBgn0053382	5SrRNA:CR33382	2546	2981	59
FBgn0053362	5SrRNA:CR33362	2545	3272	79
FBgn0053415	5SrRNA:CR33415	2519	2979	57
FBgn0053376	5SrRNA:CR33376	2505	3163	69
FBgn0053377	5SrRNA:CR33377	2494	3198	59

Table S2

HepG2 exRNA (*H.s.*)			A431 exRNA (*H.s.*)		
Gene ID	Name	Biotype	Gene ID	Name	Biotype
ENSG00000265150	RN7SL2	misc_RNA	ENSG00000265150	RN7SL2	misc_RNA
ENSG00000258486	RN7SL1	misc_RNA	ENSG00000258486	RN7SL1	misc_RNA
ENSG00000201098	RNY1	misc_RNA	ENSG00000259001	RPPH1	lincRNA
ENSG00000258486	RN7SL1	misc_RNA	ENSG00000269900	RMRP	lincRNA
ENSG00000259001	RPPH1	lincRNA	ENSG00000199990	VTRNA1-1	misc_RNA
ENSG00000266037	RN7SL3	misc_RNA	ENSG00000222328	RNU2-2P	snRNA
ENSG00000198886	MT-ND4	mRNA	ENSG00000200156	RNU5B-1	snRNA
ENSG00000198712	MT-CO2	mRNA	ENSG00000202538	RNU4-2	snRNA
ENSG00000202198	RN7SK	misc_RNA	ENSG00000202198	RN7SK	misc_RNA
ENSG00000207142	Y_RNA	misc_RNA	ENSG00000263934	SNORD3A	snoRNA

D17 exRNA (*D.m.*)			S2R+ exRNA (*D.m.*)		
Gene ID	Name	Biotype	Gene ID	Name	Biotype
FBgn0003938	snRNA:U5:63BC	snRNA	FBgn0082973	snoRNA:Psi28S-3308	snoRNA
FBgn0085364	CR34335	lincRNA	FBgn0085364	CR34335	lincRNA
FBgn0058469	CR40469	lincRNA	FBgn0086659	snoRNA:Psi18S-176	snoRNA
FBgn0085742	CR40546	lincRNA	FBgn0086667	snoRNA:Psi28S-3342	snoRNA
FBgn0086659	snoRNA:Psi18S-176	snoRNA	FBgn0002622	RpS3	mRNA
FBgn0086667	snoRNA:Psi28S-3342	snoRNA	FBgn0083057	snoRNA:Psi18S-110	snoRNA
FBgn0065098	RNaseMRP:RNA	lincRNA	FBgn0000556	Ef1alpha48D	mRNA
FBgn0082973	snoRNA:Psi28S-3308	snoRNA	FBgn0058469	CR40469	lincRNA
FBgn0083057	snoRNA:Psi18S-110	snoRNA	FBgn0261602	RpL8	mRNA
FBgn0083040	snoRNA:Psi18S-1854c	snoRNA	FBgn0066084	RpL41	mRNA

Table S3

HepG2 exRNA (*H.s.*)		A431 exRNA (*H.s.*)		D17 exRNA (*D.m.*)		S2R+ exRNA (*D.m.*)	
Name	Coordinates	Name	Coordinates	Name	Coordinates	Name	Coordinates
THE1C-int7700	chr5: 71,145,492-71,146,744	THE1C-int7700	chr5: 71,145,492-71,146,744	TART	chrX: 15,069-26,164	Copia_I-int39252	chr2L: 22,201,683-22,206,275
HERVK-int980	chr2: 230,045,354-230,045,487	L1PA227648	chrX: 108,297,820-108,302,024	(AT)n	chrX: 3,439,872-3,439,921	DIVER2_I-int16214	chr3R: 1,283,864-1,286,948
7SLRNA2870	chr14: 50,329,267-50,329,567	HERVK-int980	chr2: 230,045,354-230,045,487	(CAAT)n	chr2R: 10,245,859-10,245,899	TART57386	chrX: 15,069-26,164
7SLRNA2827	chr14: 50,053,297-50,053,597	HERVK-int25623	chr19: 36,063,339-36,066,505	(T)n	chr3R: 19,555,771-19,555,805	Gypsy2-LTR_DM969	chr2L: 22,427,715-22,428,019
L1PA227648	chrX: 108,297,820-108,302,024	7SLRNA2827	chr14: 50,053,297-50,053,597	Copia_I-int	chr3L: 2,108,581-2,113,175	TC1-2_DM11645	chr2R: 1,650,689-1,652,229
tRNA-Lys-AAG558	chr19: 36,066,749-36,066,825	tRNA-Lys-AAG558	chr19: 36,066,749-36,066,825	Copia_I-int	chr2L: 20,159,291-20,163,993	(CAG)n318	chr3R: 26,032,306-26,032,414
MER21C370	chr1: 91,852,712-91,852,785	7SLRNA2870	chr14: 50,329,267-50,329,567	(CA)n	chrX: 16,147,995-16,148,061	DM297_I-int35675	chr3R: 11,066,820-11,070,826
L1PA218775	chr5: 71,146,942-71,150,001	L1PA101524	chr21: 9,827,578-9,827,850	Copia_I-int	chr2L: 11,563,967-11,568,561	SAR_DM2740	chr3R: 25,142,562-25,144,328
L1PA101524	chr21: 9,827,578-9,827,850	L1PA218775	chr5: 71,146,942-71,150,001	Copia_I-int	chr2L: 17,820,252-17,824,847	(AT)n18	chrX: 3,439,872-3,439,921
L1PA325725	chr1: 145,277,249-145,277,491	MER21C370	chr1: 91,852,712-91,852,785	Copia_I-int	chr2L: 14,804,210-14,808,803	OSVALDO_I-int617	chr3R: 1,451,474-1,451,728

Table S4

HepG2 exRNA (*H.s.*)		A431 exRNA (*H.s.*)	
Transcript ID	Name	Transcript ID	Name
ENSG00000198886	MT-ND4	ENSG00000198938	MT-CO3
ENSG00000198712	MT-CO2	ENSG00000198886	MT-ND4
ENSG00000198804	MT-CO1	ENSG00000198712	MT-CO2
ENSG00000198938	MT-CO3	ENSG00000212907	MT-ND4L
ENSG00000087086	FTL	ENSG00000198899	MT-ATP6
ENSG00000212907	MT-ND4L	ENSG00000198804	MT-CO1
ENSG00000198899	MT-ATP6	ENSG00000198888	MT-ND1
ENSG00000198888	MT-ND1	ENSG00000228253	MT-ATP8
ENSG00000228253	MT-ATP8	ENSG00000198727	MT-CYB
ENSG00000198786	MT-ND5	ENSG00000034510	TMSB10

D17 exRNA (*D.m.*)		S2R+ exRNA (*D.m.*)	
Transcript ID	Name	Transcript ID	Name
FBgn0033926	Arc1	FBgn0002622	RpS3
FBgn0066084	RpL41	FBgn0000556	Ef1alpha48D
FBgn0261619	pAbp	FBgn0261602	RpL8
FBgn0003274	RpLP2	FBgn0066084	RpL41
FBgn0000042	Act5C	FBgn0039757	RpS7
FBgn0032987	RpL21	FBgn0032987	RpL21
FBgn0013674	mt:CoI	FBgn0000559	Ef2b
FBgn0013675	mt:CoII	FBgn0035422	RpL28
FBgn0001233	Hsp83	FBgn0026372	RpL23A
FBgn0000556	Ef1alpha48D	FBgn0023170	RpL39

Table S5

HepG2 exRNA (*H.s.*)		A431 exRNA (*H.s.*)	
GO term	FDR q-value	GO term	FDR q-value
translational elongation	1.10E-69	translational elongation	4.00E-81
cytosolic ribosome	1.30E-56	cytosolic ribosome	1.30E-68
structural constituent of ribosome	1.70E-49	ribosomal subunit	1.80E-60
ribosomal subunit	4.30E-48	structural constituent of ribosome	5.20E-59
cytosolic part	2.60E-46	ribosome	1.80E-57
ribosome	1.00E-45	cytosolic part	2.20E-55
translation	1.30E-42	translation	4.00E-50
ribonucleoprotein complex	7.20E-42	ribonucleoprotein complex	2.70E-45
cytosol	1.20E-36	cytosol	1.40E-43
cytosolic small ribosomal subunit	2.10E-28	structural molecule activity	2.30E-42

D17 exRNA (*D.m.*)		S2R+ exRNA (*D.m.*)	
GO term	FDR q-value	GO term	FDR q-value
cytosolic ribosome	7.90E-64	cytosolic ribosome	1.70E-63
cytosolic part	5.20E-57	cytosolic part	2.90E-59
structural constituent of ribosome	6.00E-55	mitotic spindle elongation	9.10E-47
ribosomal subunit	2.10E-47	spindle elongation	1.40E-46
ribosome	2.90E-45	structural constituent of ribosome	1.50E-45
mitotic spindle elongation	7.40E-43	cytosolic large ribosomal subunit	1.20E-44
spindle elongation	1.00E-42	ribosomal subunit	1.60E-36
cytosolic large ribosomal subunit	9.60E-41	ribosome	1.20E-33
lipid particle	8.40E-39	lipid particle	1.00E-32
ribonucleoprotein complex	2.60E-37	mitotic spindle organization	2.10E-31

Table S6

HepG2	
Gene ID	Name
ENSG00000213741	RPS29
ENSG00000156508	EEF1A1
ENSG00000163631	ALB
ENSG00000084674	APOB
ENSG00000130167	TSPAN16

D17			
Gene ID	Name	Gene ID	Name
FBgn0034770	Obp58d	FBgn0052774	Muc4B
FBgn0005640	Eip63E	FBgn0038919	CG17843
FBgn0011288	Snap25	FBgn0000721	for
FBgn0262098	CG42851	FBgn0038508	CG5866
FBgn0052214	CG32214	FBgn0030926	psh
FBgn0040950	Muc26B	FBgn0035436	CG12016
FBgn0032313	CG14070	FBgn0038449	CG17562
FBgn0032851	CG13970	FBgn0036226	CG7252
FBgn0038078	CG14391	FBgn0038979	CG7046
FBgn0085488	CG34459	FBgn0063485	Lasp
FBgn0053554	Nipped-A	FBgn0263780	CG17684
FBgn0038733	CG11407		
FBgn0264395	CG43843		
